# BioClock—optimizing Bright Light Therapy for adults with depression: a study protocol for a multicenter randomized clinical trial on treatment strategies, response predictors, and chronobiological and neurobiological mechanisms

**DOI:** 10.1186/s13063-025-08984-7

**Published:** 2025-10-14

**Authors:** E. Visser, O.G. Rus-Oswald, A.J.W. van der Does, M.C.M. Gordijn, M.C. Marcelis, Y.A.W. de Kort, P.P. Oomen, L.J.M. Schlangen, T. Seetsen, C.J.P. Simons, N. Antypa

**Affiliations:** 1 Human Technology Interaction group, Department of Industrial Engineering and Innovation Sciences, Eindhoven University of Technology, Den Dolech 2, 5612 AZ, Eindhoven, The Netherlands; 2 Department of Clinical Psychology, Faculty of Social Sciences, Leiden University, Wassenaarseweg 2, 2333 AK, Leiden, The Netherlands; 3 Leids Universitair Behandel- en Expertise Centrum, Sandifortdreef 17, Leiden, 2333 ZZ The Netherlands; 4 Chrono@Work B.V., Friesestraatweg 213, Groningen, 9743 AD The Netherlands; 5 Chronobiology Unit, Groningen Institute for Evolutionary Life Sciences, University of Groningen, Nijenborgh 7, Groningen, 9747 AG The Netherlands; 6 GGzE Mental Health Institute of Eindhoven and the Kempen, Eindhoven, 5626 ND Dokter Poletlaan 40, The Netherlands; 7 Department of Psychiatry and Neuropsychology, Mental Health and Neuroscience Research Institute, Maastricht University, Minderbroedersberg 4-6 , Maastricht, 6211 LK The Netherlands; 8 Behavioural Science Institute, Radboud University, Nijmegen, The Netherlands

**Keywords:** Bright Light Therapy, Chronobiology, Depression, Mood disorders, Melatonin, Sleep, Ecological Momentary Assessment, Magnetic resonance imaging

## Abstract

**Background:**

Bright Light Therapy (BLT) is an effective treatment for various mood disorders, but challenges remain in optimizing its real-world administration, predicting individual responses, and understanding its mechanisms.

**Methods:**

This multicenter randomized controlled trial will enroll 231 patients with unipolar or bipolar depression, all currently experiencing a depressive episode. Participants will be randomized to one of three BLT interventions: (1) traditional home-based BLT, (2) BLT administered in the *LightCafé*—a novel setting promoting social interaction and lifestyle support, or (3) *LightCafé* BLT with personalized timing of both light and darkness by adding the use of blue light blocking glasses. The treatment duration will range from one to three weeks, based on preset definitions of participant’s response. The primary outcome is the change in depressive symptoms, assessed by a blinded rater. Secondary outcomes include subjective and objective sleep measures, circadian rhythm parameters from melatonin patterns and actigraphy, and functional outcomes. Change processes will be assessed using ecological momentary assessments of daily affect and energy levels, light exposure sensors, and via brain imaging.

**Discussion:**

This trial is the first to directly compare different BLT administration models, including a *LightCafé* setting. It aims to identify predictors of BLT response, investigate chronobiological and neurobiological mechanisms, and provide insights to optimize BLT for diverse patient populations. The results will inform clinical guidelines and enhance BLT's accessibility and effectiveness.

**Trial registration:**

Registered on July 14, 2023, at clinicaltrials.gov under number NCT05958940.

**Supplementary Information:**

The online version contains supplementary material available at 10.1186/s13063-025-08984-7.

## Administrative information

Note: the numbers in curly brackets in this protocol refer to SPIRIT checklist item numbers. The order of the items has been modified to group similar items (see http://www.equator-network.org/reporting-guidelines/spirit-2013-statement-defining-standard-protocol-items-for-clinical-trials/).

**Table:** 

Title {1}	**BioClock—Optimizing Bright Light Therapy for Adults with Depression:** A Study Protocol for a Multicenter Randomized Clinical Trial on Treatment Strategies, Response Predictors, and Chronobiological and Neurobiological Mechanisms
Trial registration {2a and 2b}.	The trial is registered on July 14, 2023, at clinicaltrials.gov under number NCT05958940; https://clinicaltrials.gov/study/NCT05958940
Protocol version {3}	Protocol version 3.1, 13–10-2023 Revision Chronology: • 13–10-2023 – version 3.0 Final protocol version after Ethical Approval • 12–05-2023 version 2.0 First submitted version for Ethical Approval • 23–12-2022 – version 1.0 Initial Draft version
Funding {4}	This research is part of the BioClock project (project number: 1292.19.077) of the research program National Science Agenda: Research on Routes by Consortia (NWA-ORC), which receives (partial) funding from the Netherlands Organization for Scientific Research (NWO).
Author details {5a}	**Emma Visser** (e.visser1@tue.nl) – corresponding author *Human Technology Interaction group,* *Department of Industrial Engineering and Innovation Sciences,* *Eindhoven University of Technology* *Den Dolech 2, 5612 AZ Eindhoven, The Netherlands* **Georgiana Oana Rus-Oswald** (o.g.rus@fsw.leidenuniv.nl) *Department of Clinical Psychology,* *Faculty of Social Sciences,* *Leiden University* *Wassenaarseweg 52, 2333 AK Leiden, The Netherlands* ***A.J. W. van der Does ****(vanderdoes@fsw.leidenuniv.nl)* *Leids Universitair Behandel- en Expertise Centrum* *Sandifortdreef 17 2333 ZZ Leiden, the Netherlands* ***Marijke C.M. Gordijn ****(marijke.gordijn@chronoatwork.com)* *Chrono@Work B.V., Groningen, The Netherlands* *Friesestraatweg 213, 9743 AD Groningen, the Netherlands* *&* *Chronobiology Unit,* *Groningen Institute for Evolutionary Life Sciences,* *University of Groningen* *Nijenborgh 7 9747 AG Groningen, the Netherlands* ***Machteld M.C. Marcelis****(Machteld.Marcelis@ggze.nl)* *GGzE Mental health institute of Eindhoven and the Kempen,* *Dokter Poletlaan 40, 5626ND, Eindhoven, the Netherlands* *&* *Department of Psychiatry and Neuropsychology,* *Mental Health and Neuroscience Research Institute,* *Maastricht University* *Minderbroedersberg 4–6 6211 LK Maastricht, The Netherlands* ***Yvonne. A.W. de Kort ****(Y.a.w.d.Kort@tue.nl)* *Human Technology Interaction group,* *Department of Industrial Engineering and Innovation Sciences,* *Eindhoven University of Technology* *Den Dolech 2, 5612 AZ Eindhoven, The Netherlands* ***Priscilla P. Oomen****(priscilla.oomen@ru.nl)* *Behavioural Science Institute,* *Radboud University, Nijmegen,* *Houtlaan 4 6525 XZ Nijmegen, The Netherlands* ***Luc J.M. Schlangen, ****(l.j.m.Schlangen@tue.nl)* *Human Technology Interaction group,* *Department of Industrial Engineering and Innovation Sciences,* *Eindhoven University of Technology* *Den Dolech 2, 5612 AZ Eindhoven, The Netherlands* ***Tanja Seetsen ****(tanja.seetsen@ggze.nl)* *GGzE Mental health institute of Eindhoven and the Kempen,* *Dokter Poletlaan 40, 5626ND, Eindhoven, the Netherlands* ***Claudia J.P. Simons ****(Claudia.simons@ggze.nl* *GGzE Mental health institute of Eindhoven and the Kempen,* *Dokter Poletlaan 40, 5626ND, Eindhoven, the Netherlands* *&* *Department of Psychiatry and Neuropsychology,* *Mental Health and Neuroscience Research Institute,* *Maastricht University* *Minderbroedersberg 4–6 6211 LK Maastricht, The Netherlands* ***Niki Antypa ****(NAntypa@fsw.leidenuniv.nl)* *Department of Clinical Psychology,* *Faculty of Social Sciences,* *Leiden University* *Wassenaarseweg 52, 2333 AK Leiden, The Netherlands*
Name and contact information for the trial sponsor {5b}	Leiden University Wassenaarseweg 52, 2333 AK Leiden, The Netherlands
Role of sponsor {5c}	This funding source had no role in the design of this study and will not have any role during its execution, analyses, interpretation of the data, or decision to submit results.

## Introduction

### Background and rationale {6a}

Depressive symptoms place a heavy burden on mood, motivation, and everyday functioning. Yet conventional antidepressants often require weeks to exert their full effects and can burden patients with persistent side effects. Bright Light Therapy (BLT)—which involves repeated exposure to a high-intensity light source—offers a compelling alternative: rapid relief, robust antidepressant impact, and only minimal, transient discomfort. In fact, BLT has now earned a place in clinical guidelines and is widely used in countries across the world—albeit with varied specifications of light intensity, treatment duration, and spectral composition of the light [1–4]. Below, we review BLT’s efficacy across mood disorders, introduce an optimized delivery model, explore predictors of response, and examine the mechanisms underpinning its antidepressant action, all to introduce a multi-center randomized controlled trial (RCT) that investigates the optimization, response prediction, and chronobiological and neurobiological mechanisms of BLT.

### The efficacy of Bright Light Therapy across various mood disorders

The clearest evidence for the efficacy of BLT comes from Seasonal Affective Disorder (SAD). In one of the first trials, Rosenthal and colleagues (1984; *N* = 29) found that 30–60 min of bright light—2500 to 10,000 lx—each morning lifted mood within just 3 to 5 days [5]. Larger, placebo‐controlled studies soon confirmed these results. In a pivotal randomized controlled trial with 96 SAD patients, 1.5 h of 6000‐lux exposure per day produced remission in 61% of participants, compared with just 32% in a dim‐light control group after 3 weeks [6]. Meta‐analyses of around 20 trials (*N*≈1200) report large effect sizes (0.60–1.08), with morning administration and intensities of at least 2500 lx yielding the greatest benefit [7, 8].

BLT’s efficacy, however, extends well beyond SAD. In non‐seasonal major depressive disorder (MDD), early evidence of BLT was mixed [7], but recent meta-analyses now clearly support its use [9, 10]. A meta‐analysis of 20 RCTs (*N* = 881) found a moderate pooled effect (SMD ≈ − 0.4), again highlighting greater efficacy at higher intensities (≥ 7500 lx) and with consistent morning dosing [10]. Even in bipolar depression—where early concerns suggested morning light might trigger mania—a meta‐analysis of 11 studies (*N* = 567) showed significant reductions in depressive symptoms without any increase in manic episodes, provided BLT was used alongside mood stabilizers [11]. Beyond mood disorders, preliminary evidence points to benefits in, e.g., Attention-Deficit/Hyperactivity Disorder (ADHD) [12], Parkinson’s disease [13], dementia [14], and cancer-related fatigue [15]. Across this spectrum of indications, patients typically experience relief within 1 to 2 weeks with minimal side effects. Notable is also the synergistic effects that are achieved when combining BLT with pharmacotherapy [16, 17] underscoring its promise as a transdiagnostic adjunctive intervention.

### From efficacy to optimization

With efficacy well established the challenge shifts from demonstrating that BLT works to refining its delivery. While optimal intensity, spectral composition, and treatment duration remain open questions [9, 10], the greatest impact may come from moving beyond standalone BLT and embedding it within complementary interventions and supportive contexts that together target depressive symptoms. Conventional BLT typically confines patients to their homes or to (semi-)clinical spaces, sitting alone under a bright lamp in an otherwise sterile, low—interaction environment. Although this format reliably delivers the prescribed light dose, it overlooks the potent effects of social engagement [18, 19], behavioral activation [20], and environmental cues [21] on mood and treatment adherence that can amplify and extend BLT’s benefits—transforming it from a solitary task into an immersive, habit-forming therapy. To bring this vision to life, this trial introduces the LightCafé [22]—a BLT delivery model set in a café-style space that feels restorative rather than clinical. Here, instead of solitary lamp setups, participants receive their morning light exposure embedded within a rich psychosocial context. Daily guided social conversations with a therapist foster positive affect and counteract isolation, while lifestyle coaching—from diet and exercise to sleep hygiene and routine building—helps translate acute mood changes into durable behavioral change. This multi-faceted approach is thought to facilitate longer lasting and sustainable change in patients struggling with depressive symptoms.

An unpublished observational study by Ottenheijm and Maes reports high response rates and strong patient satisfaction among patients in the LightCafé. However, the current LightCafé implementation follows a non-personalized schedule and does not yet leverage chronobiological insights now central to depression treatment. Evidence shows that aligning light exposure with each individual’s circadian phase and minimizing evening light, is critical for consolidating sleep and stabilizing rhythms [23, 24], both of which directly support mood regulation [25]. To address this, the current trial will refine the LightCafé protocol by synchronizing morning light sessions to each participant’s sleep–wake rhythm and by providing blue-light-blocking glasses (BBG) to minimize evening light exposure. In marrying the psychosocial power of the café environment with precision chronotherapy, we aim to deliver more effective and personalized BLT.

### Predicting treatment response

The next step in optimization involves more precise therapy selection upfront, as without knowing who benefits most, we risk offering intensive BLT protocols to unlikely responders. However, to date, we lack hypothesis‐driven investigations into which patient characteristics and behaviors predict BLT response. Secondary analyses in SAD cohorts suggest that individuals presenting with atypical features—such as increased appetite, hypersomnia, and a positive diurnal mood variation—alongside higher baseline BMI tend to experience the most rapid and robust improvement with morning light exposure [26, 27]. Those exhibiting melancholic symptoms—psychomotor retardation, early and late insomnia, appetite loss, pervasive guilt, and worse mood upon awakening—often derive little benefit [26]. Similarly, in treatment-resistant inpatients undergoing combined wake and light therapy, early diurnal symptom shifts, evening chronotype, and higher educational history have been linked to both short- and long-term remission [28]. Yet it remains unknown whether these results generalize across the full spectrum of unipolar and bipolar depressions. Clarifying these predictors is crucial: it would empower clinicians to stratify patients by likelihood of response, and ultimately render BLT a more efficient, cost-effective, and patient-centered antidepressant strategy.

### The chronobiological and neurobiological mechanisms underlying BLT

Next, this study aims to examine the mechanisms by which BLT alleviates depressive symptoms, as currently no theory has conclusively explained its effects. The most widely accepted framework—the chronobiological phase-shift hypothesis—proposes that BLT realigns the circadian system with the natural 24-h light–dark cycle [29], correcting rhythm disturbances common in major depressive and bipolar disorders [30–33], and thereby alleviating depressive symptoms. Although robust phase advances following morning BLT have been reliably documented [34], attempts to link these shifts directly to reductions in depressive symptoms have yielded inconsistent results [35–40]. Several other studies further challenge a circadian explanation, suggesting that light exerts its mood-enhancing effects through neural pathways independent of the central clock [17, 41–43], or via directly altering the serotonin release [44, 45]. Consequently, while BLT is often framed as a chronotherapeutic intervention, key questions persist about the true role—and relative importance—of underlying chronobiological mechanisms which we aim to clarify in the current trial.

Light’s impact on sleep quality and energy levels may provide an additional—and potentially contrasting—route by which BLT exerts its antidepressant effects. Up to 90% of depressed patients report insomnia, delayed sleep onset, and fragmented rest at night [46, 47], while many also suffer daytime sleepiness [48]. Controlled trials show that daily morning BLT consolidates sleep, shortens sleep-onset latency, and improves alertness during the day [49, 50], but complicated by the multidirectional associations between mood, sleep, and energy levels [51–53], it remains unclear whether these changes stem from circadian stabilization, light’s direct alerting effects [54, 55], a synergistic interplay of both, or an unknown mechanism. By tracking light exposure, sleep, energy, and mood on a day-to-day level in the current trial, we aim to map how these factors interact with each other over time and gain insight into each one’s role in BLT’s antidepressant mechanism.

Neuroimaging adds a third dimension: though sparse, early studies hint that BLT engages both emotion-processing hubs (amygdala, habenula) and circadian centers (suprachiasmatic nucleus, hypothalamus) [41, 42, 56–58]. Some RCT studies have evaluated the effects of BLT alone or in combination with other chronotherapeutics (e.g., sleep deprivation) in MDD [59, 60], subthreshold depression in adults [57, 61], in young adults [62, 63], or bipolar depression [64, 65]. They used mostly functional neuroimaging techniques to evaluate brain connectivity at rest and reported that weeks (varying between 3 and 8 weeks) of morning BLT restore connectivity within default-mode, frontoparietal, and salience networks—and that these neural changes predicted and correlated with symptom improvement [57, 61, 62, 64]. Interestingly, these studies bring some indications about the use of these neural parameters as potential biomarkers for disease in such that the neural changes found were linked to specific neurotransmitter systems (amongst others serotonin, dopamine) [62], but also to metabolic changes (glutamate/glutamine) [65]. One needs to mention that these studies have a high heterogeneity in study design and sample type used but also in neuroimaging data analysis approach, i.e., some using specific regions of interest and not the whole brain, i.e., amygdala [60], cingulate cortex [62], and cerebellum [61]. Hence, a clear mechanistic model is hard to determine at this stage. In addition, besides evident functional changes after BLT, there are also structural change indications after BLT administration in, e.g., posttraumatic stress disorder (PTSD) [66] and mild traumatic brain injury (mTBI) cohorts [67, 68]. The existing studies suggest that blue-light exposure can increase gray-matter volume and white-matter integrity [66–68].

In the current study, we therefore plan to take a multimodal neuroimaging approach by combining both functional and structural change analysis as well as going from whole brain analysis to specific regions of interest and link it to symptom change pre-post BLT. Hence, we believe that this approach will give a more comprehensive mechanistic model of the neural pattern behind BLT in depression. Furthermore, no study to date has combined precise circadian profiling, sleep metrics, and multimodal imaging in a large, multicenter sample and using the light cafe approach which brings the lacking social component into the depression model—a gap our trial is uniquely poised to fill.

### Objectives {7}

The primary objective of this study is to comprehensively assess and compare the effectiveness of three distinct BLT approaches for the treatment of depressive symptoms: (1) standard home-based BLT (*Light@Home)*, (2) BLT at the *LightCafé (LightCafé)*, and (3) BLT at the *LightCafé* with personalized timing of light and darkness using BBG (*LightCafé* +).

Secondary objectives are:

i. The identification of predictors of treatment response, encompassing baseline clinical characteristics, sleep metrics, circadian parameters, and light-related behaviors.
ii. Elucidation of underlying mechanisms of BLT’s efficacy by investigating the contribution of circadian rhythm changes, enhancing our understanding of the complex interactions between mood, energy levels, and sleep within the context of BLT and exploration of the neural effects of BLT by studying both functional and structural brain changes before and after treatment.

Importantly, we are not testing BLT’s efficacy for specific diagnostic categories; rather, we ask whether embedding BLT within a rich psychosocial setting and personalizing its timing can amplify and prolong its antidepressant effect, all while unraveling who benefits most and through which pathways. By moving from “Does BLT work?” to “How can we make it work better, for whom, and why?”, this multicenter RCT will lay the groundwork for personalized and embedded BLT protocols and real-world clinical guidelines.

### Trial design {8}

This study constitutes a multi-center, single-blind randomized controlled trial assessing the effects of BLT for individuals with a current depressive episode (either diagnosed with SAD, MDD, or bipolar disorder). The study consists of three phases—the baseline phase, the treatment phase, and the follow-up phase. During the treatment phase, participants will receive BLT through one of three administration strategies—*Light@Home*,* LightCafé*, or* LightCafé* + —with treatment lasting 1 to 3 weeks, depending on their individual response. Baseline assessments occur 1 week prior to treatment, with an additional week for participants undergoing melatonin assessments. Follow-up evaluations occur at 4 weeks, 10 weeks, and 4 months after the start of the treatment.

## Methods: participants, interventions, and outcomes

### Study setting {9}

BLT is administered either at home or in the *LightCafé*, depending on the treatment condition. *LightCafé* locations are distributed across two mental healthcare institutions in the following way:

Mental Health Clinic of Eindhoven and the Kempen (GGzE) in Eindhoven

- *LightCafé* Ketelhuis Boschdijk, Eindhoven
- LightCafé Lucifer Kennedyplein, Eindhoven
- *LightCafé* Library Veldhoven, Veldhoven

Leids Universitair Behandel- en Expertise Centrum (LUBEC) in Leiden.

- *LightCafé *Faculty of Social Sciences, Leiden

### Eligibility criteria {10}

#### General eligibility criteria

Eligibility will be assessed during the intake by study team members trained in administering the MINI International Neuropsychiatric Interview 5.0.0 (M.I.N.I.; [69]). The inclusion criteria are as follows:

- Age between 18 and 65.
- Diagnosis of unipolar or bipolar depression (seasonal or non-seasonal) confirmed by the M.I.N.I. [69].
- Currently experiencing a depressive episode, as indicated by a Quick Inventory of Depressive Symptomatology Self-Report (QIDS-SR; [70]) score of 6 or higher.
- Proficiency in either Dutch or English is required for questionnaire completion.

Participants are excluded from participation if they:

- Are currently experiencing a (hypo)manic or mixed episode, as determined by the M.I.N.I. assessment [69].
- Are presently in a psychotic episode, as determined by the M.I.N.I. assessment [69].
- Exhibit significant active suicidality, indicated by a score of 10 or higher on the M.I.N.I. module [69].
- Initiated antidepressant therapy (including medication, psychotherapy, BLT, or other specific depression treatments) within the last 2 months prior to study enrolment.
- Have a bipolar disorder but are currently not under mood-stabilizing treatment for at least 1 month at the recommended dosage.
- Have used melatonin or agomelatine in the past month.
- Are currently using antibiotics.
- Are currently taking medications that increase light sensitivity.
- Have travelled across more than one time zone in the past month or during the treatment period.
- Have travelled to sunny holiday destinations or engaged in outdoor winter sports in the past month.
- Have pre-existing eye and skin disorders such as retinitis pigmentosa, porphyria, chronic actinic dermatitis, and sun-induced urticaria.
- Have systemic disorders with potential retinal involvement, such as rheumatoid arthritis and systemic lupus erythematosus.
- Have color blindness (based on self-report).
- Worked night shifts within the last three months.
- Suffer from (retinal) blindness, severe cataracts, or glaucoma.
- Experience light-induced migraine or epilepsy.
- Are currently pregnant or have a child Younger than 18 months old.

#### MRI Assessment Criteria

For participants undergoing Magnetic Resonance Imaging (MRI) assessments, additional criteria apply:

- Individuals with claustrophobia, a history of neurological disorders, or foreign non-MRI compatible metal objects in or on their body are ineligible for MRI assessments.

Participants not meeting the MRI eligibility criteria can still participate in the broader trial, provided they meet the general participation criteria.

#### Eligibility of the research team

BLT will be administered by trained clinical professionals, including psychologists, nurses, and social workers, with assistance from master’s-level students. The assessment of the primary outcome will be conducted by blind raters, who will either be psychologists or master’s-level psychology students.

#### Who will take informed consent? {26a}

All patients referred to BLT at the study centers are invited to participate in the study. Those expressing interest receive information via email. A follow-up phone call is conducted to confirm their interest and schedule an intake appointment. During the intake, a member of the study team goes over the information letter and informed consent document in detail and answers any questions. If a patient decides to participate, they sign the informed consent and proceed with the diagnostic interview.

#### Additional consent provisions for collection and use of participant data and biological specimens {26b}

In addition to providing consent for participation in the trial, participants are offered the opportunity to grant separate consent for specific aspects related to their data and biological specimens. These supplementary consent provisions encompass:

- The permission for data retention for a minimum of 15 years and the potential use in future research
- The choice to allow or prohibit recording of clinical interviews,
- The decision to participate or abstain from saliva collection, and
- The consent that the brain scan is not a medical sequence, but if an incidental finding is detected (any brain abnormality), the hospital will inform the general practitioner and the patient.

Participants may accept or decline these provisions without any impact on their eligibility for the trial, thus ensuring participants’ autonomy in the utilization of their data and specimens.

## Interventions

### Explanation for the choice of comparators {6b}

This study compares the effectiveness of three distinct BLT administration strategies for treating depression:

I. **Home-Based BLT (*Light@Home*)**: Patients receive BLT in their own homes.
II. **BLT in LightCafé (*LightCafé*)**: Patients undergo BLT in a café-like environment (*LightCafé*), which emphasizes lifestyle improvements alongside the therapy.
III. **Personalized BLT in the LightCafé (*LightCafé +*)**: Patients receive BLT in the *LightCafé*, with treatment timing tailored to their individual sleep–wake pattern and personalized timing of darkness with the use of BBG.

The *Light@Home* condition serves as an active control condition against which both other conditions will be evaluated. Given that our primary objective is not to measure the effectiveness of BLT in alleviating depressive symptoms per se, but rather to optimize its delivery, the inclusion of a placebo or treatment-as-usual condition in this trial is deemed unwarranted. To assess and possibly control for the difference in expectations of treatments, all participants will complete the Credibility/Expectancy Questionnaire (CEQ; [71]) before treatment onset.

### Intervention description {11a}

In all study arms, participants are subjected to BLT in adherence to Dutch BLT guidelines [72], utilizing an Innolux LED light Lamp delivering light with an illuminance of 10,000 lx at a distance of 70 cm and a color temperature of 3800 K. BLT sessions, lasting 30 min each, are scheduled from Monday to Friday between 7:30 AM and 10:30 AM.

The number of BLT sessions is contingent upon the individual response of each participant, with a maximum duration of 3 weeks. Initially, participants undergo five consecutive BLT sessions during the workweek. Subsequently, the therapeutic efficacy is evaluated utilizing the QIDS-SR during the Friday session. If the QIDS-SR falls below 6, remission is inferred, leading to the termination of BLT. Alternatively, an additional five sessions are administered. This evaluation and decision process is repeated at the end of the second week. If the QIDS-SR score is still above 6, a third and final week of treatment is provided.

This individualized strategy is intended to reduce the small risk of (hypo)manic switches, which occur in less than 2% of bipolar patients and almost exclusively in those not maintained on mood stabilizers [11, 73]. We will continuously monitor for (hypo)manic symptoms using the Young Mania Rating Scale (YMRS; [74]) and clinical observation: a YMRS score ≥ 13 and clinician‐identified symptoms will lead to immediate treatment cessation, while scores of 10–11 will prompt a joint review with a second clinician to decide on discontinuation [75, 76].

### The LightCafé Approach

**Fig. 1:**
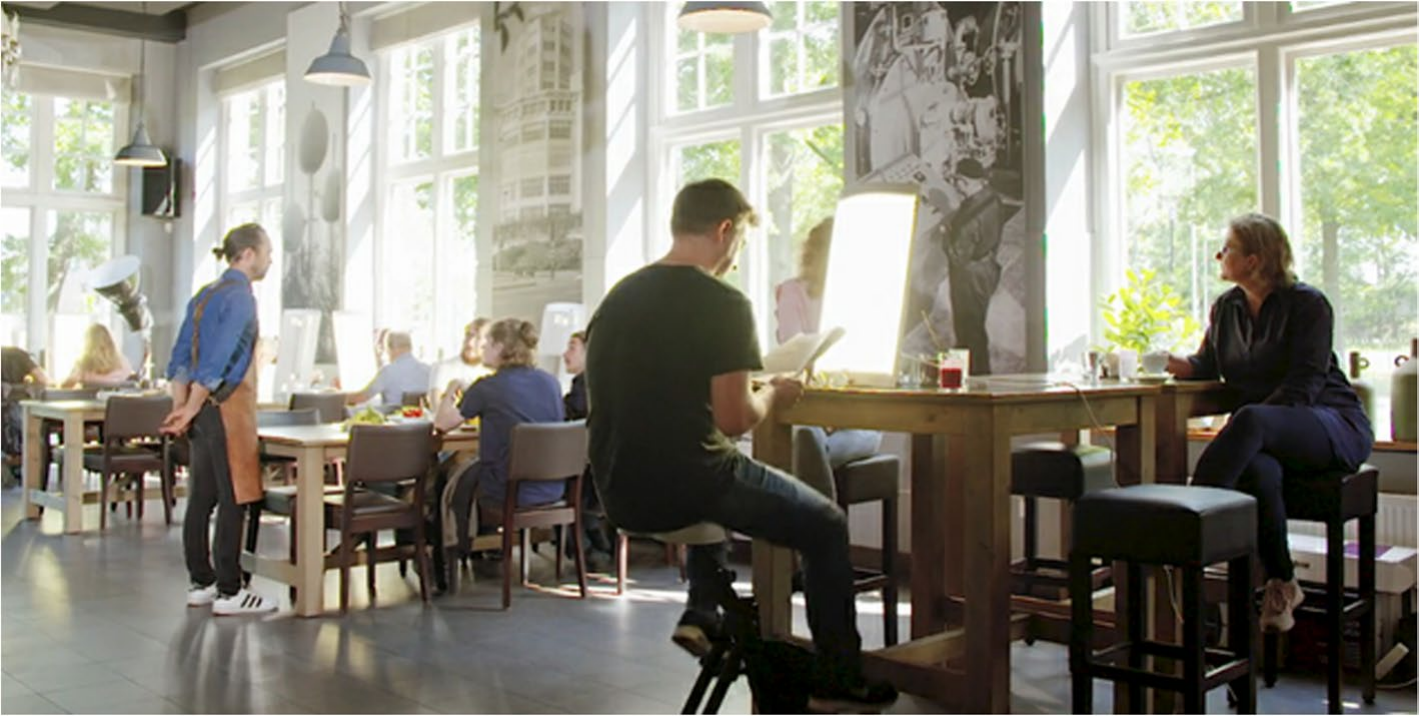
The LightCafé is a café-style space where each participant receives BLT against a backdrop of restorative design elements (e.g., ample natural light, greenery, artwork, and clustered seating). Sessions include 5–10 min of one-on-one lifestyle coaching with professional and take-home workbooks for self-guided sleep-, exercise-, and nutrition-support. All trial participants in the LightCafé and LightCafé+ arms are treated in this setting. Photo: Boschdijk location, GGzE Eindhoven en de Kempen, Netherlands

In the *LightCafé* and *LightCafé* + conditions, BLT is administered in the *LightCafé*. This café-like environment is intentionally designed to provide a comfortable, informal, and welcoming atmosphere, in stark contrast to traditional clinical environments. The interior design of *LightCafé* emphasizes restorative elements, with soothing colors, comfortable seating, and natural light exposure, while the layout encourages social interaction among patients. Various decor elements, such as plants and artwork, contribute to a warm and inviting environment that enhances overall well-being. Figure 1 provides a visual representation of *LightCafé*.

Each BLT session in the *LightCafé* includes 5–10-min consultations with a professional nurse or psychologist, during which participants discuss various aspects of their lifestyle, such as sleep hygiene, exercise, nutrition, daily routines, social interaction, and overall healthy living. These brief consultations are integrated into the light therapy sessions to provide personalized guidance and support. Additionally, participants are provided with workbooks designed for independent use, enabling them to actively work on and improve their lifestyle habits outside of the sessions. The ultimate goal of the *LightCafé* approach is to foster a stable rest-activity pattern, promote a more active lifestyle, ensure a fulfilling daily routine, and encourage social interaction, all within a treatment environment that enhances the overall treatment experience.

### Personalized chronotherapeutic strategies

In the *LightCafé* + arm, BLT timing is personalized, and participants wear BBG according to their individual sleep–wake rhythms. These adjustments aim to promote sleep regularity and synchronize sleep phase with the endogenous biological night. Before commencing treatment, each participant’s current sleep onset time (CSO) is established based on their baseline sleep data obtained from the Consensus Sleep Diary (CSD; [60]. Following this, a target sleep onset time (TSO) is collaboratively determined through a phone call with a member of the study team. During this conversation, the participant’s daily routine, social and work obligations, and personal preferences for sleep onset and offset are discussed. The aim is to establish a sleep schedule that accommodates approximately 7–8 h of nightly sleep and fits well within the participant’s everyday life.

Based on the relation between CSO and TSO, one of the following timing strategies is employed:

- *Stabilize*: When CSO is within 30 min of TSO, the aim is to not shift sleep timing and the circadian clock. Morning BLT is administered approximately 11 h after TSO, and participants wear BBG during the last hour before TSO.
- *Advance*: When CSO occurs more than 30 min later than TSO, the aim is to advance the sleep timing and the biological clock by exposing patients to BLT earlier in the morning (around 8–9 h after CSO) and having them wear BBG during the last 2 h before CSO.
- *Delay*: In cases where CSO is more than 30 min earlier than TSO, a delay in sleep–wake cycles and the biological clock is the goal. Morning BLT is administered later in the morning (around 11 h after CSO), and BBG are worn in the morning, during the first 2 h after awakening.

The chronobiological strategy is selected and implemented on a weekly basis, based on a reassessment of the CSO, using CSD entries from that week, and the TSO, determined via phone contact. Treatment times will always remain within the 7:30 AM to 10:30 AM window. This time frame was chosen because morning light has been shown to be most effective in reducing depressive symptoms [7]. While some individuals may theoretically benefit from alternative timings, individual adjustments are limited by the practical constraints of the clinical setting. If the prescribed strategy suggests a time outside this window, the closest suitable time within the range is selected.

### Criteria for discontinuing or modifying allocated interventions {11b}

Participants can withdraw from the study at any time without providing a specific reason. BLT is discontinued upon the patient achieving remission or exhibiting mania symptoms as per the YMRS or clinical assessment. Furthermore, treatment discontinuation can be initiated by clinical staff when they consider it to be in the best interest of the patient.

### Strategies to improve adherence to interventions {11c}

Treatment adherence is assessed using two complementary methods: EMA and personal light exposure sensors. Each day, participants receive a smartphone questionnaire asking whether they adhered to the light therapy protocol. If not, they are prompted to indicate the reason for non-compliance. In parallel, participants wear personal light exposure sensors throughout the study period to objectively record light exposure and verify whether the prescribed timing and dosage were followed.

To support protocol adherence and increase participant engagement, a feedback session is scheduled after the post-treatment assessment. During this session, participants receive personalized feedback based on their individual data (e.g., daily affect changes, and actigraphy reports) collected during the intervention.

### Relevant concomitant care permitted or prohibited during the trial {11d}

Participants are allowed to continue existing antidepressant treatments alongside BLT, provided these therapies—pharmacological or non-pharmacological—were initiated at least 2 months prior to study entry. This includes antidepressant medications, psychotherapy, EMDR, or other interventions aimed at reducing depressive symptoms. While the initiation of new treatments or dosage adjustments during the study period is discouraged, they are not prohibited. Any such changes will be carefully documented and accounted for in the statistical analysis to control for potential confounding effects.

### Provisions for post-trial care {30}

In the event of a successful treatment, the opportunity for another round of BLT becomes available 3 months after the last treatment session, provided there are no clinical contraindications, and the final follow-up of the study has been completed. If the treatment is considered unsuccessful, official communication will be sent to the referring healthcare provider, and the responsibility for further treatment will rest with them.

Participants in this study are not provided with research subject insurance coverage, as BLT is a non-invasive treatment associated with a low likelihood of mild and short-term side effects.

### Outcomes {12}

#### Primary study outcome—clinical improvement in depressive symptoms

The primary outcome of this study is the clinical improvement in depressive symptoms assessed with the Montgomery Åsberg Depression Rating Scale (MADRS; [78]) from baseline to post-treatment. Independent raters, unaware of participants’ treatment conditions and assessment points, will conduct MADRS evaluations. Other key metrics based on the MADRS include remission (participants with post-treatment scores of 10 or lower [79]) and response rates (participants with 50% reduction in depressive symptoms from baseline to post-treatment assessment).

### Secondary study outcomes

We assess a range of secondary outcomes, categorized as mood-related and functional outcomes, sleep-related outcomes, circadian parameters, brain imaging parameters, and light-related parameters.

## Mood-related and functional outcomes

Complementary to the clinician-rated depressive symptoms, self-reported depressive symptoms will be evaluated using the QIDS-SR, administered weekly from baseline to post-treatment, as well as at the 10-week and 4-month follow-ups. In addition to tracking self-reported depression scores over time, we will utilize QIDS-SR data to assess self-rated remission rates, self-rated response rates, time to remission, and relapse rates.

Momentary affect measures are evaluated using EMA to assess mood throughout the day. From the baseline week through 1 week after treatment, participants receive eight daily smartphone alerts containing items from the Positive and Negative Affect Scale [80] the Activation-Deactivation Adjective Check List [81] and additional items adapted specifically for this EMA. Momentary Positive Affect (PA) is assessed using the average score of three items in the EMA: “I feel cheerful,” “I feel content,” and “I generally feel well,” each rated on a 7-point scale from 1 (*not at all*) to 7 (*very*). Momentary Negative Affect (NA) is evaluated using the average of six items: “I feel irritated,” “I feel insecure,” “I feel lonely,” “I feel anxious,” “I feel down,” and “I feel guilty,” rated similarly. Diurnal mood variations will be assessed by comparing momentary affect states in the morning with momentary affect in the afternoon and evening.

Functional outcomes will be evaluated using the Short Form Health Survey (SF-12; [82]), the Mental Health Quality of Life Questionnaire (MHQoL; [83]), at baseline, post-treatment, and during follow-up, providing us with parameters for overall functioning and quality of life, respectively.

## Sleep-Related Outcomes

Subjective sleep metrics are collected using a short version of the CSD which is administered daily during the baseline, treatment, and first follow-up week through the use of EMA (morning prompt). Key metrics derived from this diary include sleep duration, sleep onset, sleep onset latency, sleep offset, midsleep timing, wake after sleep onset, and sleep quality. Concurrently, objective sleep metrics are obtained from actigraphy data collected using the MotionWatch8 (CamNtech, Cambridge, UK) during the baseline, treatment, and first week post-treatment. Objective sleep parameters mirror those of the subjective measures and include sleep duration, sleep onset, sleep onset latency, sleep offset, midsleep timing, wake after sleep onset, sleep fragmentation, and sleep efficiency.

Momentary Energy Levels are evaluated using the average score on the EMA items “I feel energetic,” “I feel active,” and “I feel lively,” and the additional question “how alert or sleepy do I feel right now?” rated from 1 (*extremely sleepy*) to 9 (*extremely alert*). Additionally, subjective sleep quality and insomnia severity are assessed using the Pittsburgh Sleep Quality Index (PSQI; [84]) and the Insomnia Severity Index (ISI; [85]), administered at baseline, post-treatment, and during the 10-week and 4-month follow-ups.

## Circadian rhythm parameters

Dim Light Melatonin Onset (DLMO) is evaluated at baseline and post-treatment as the gold standard for determining circadian phase. This assessment involves collecting eight saliva samples around habitual sleep onset. DLMO was defined as the time when melatonin concentrations exceeded the 3 pg/mL threshold upon linear interpolation of subsequent melatonin values and continued to rise. Additionally, we assess the phase angle difference between DLMO and sleep onset (based on actigraphy), a crucial metric for evaluating circadian misalignment. We also analyze actigraphy-derived metrics, including the least active 5-h period (L5), the most active 10-h period (M10), and the difference in activity between L5 and M10, which serves as a proxy for amplitude. Other metrics derived from actigraphy include rest-activity periodicity, inter-daily stability, and intra-daily variability. Furthermore, we evaluate chronotype using the Morningness-Eveningness Questionnaire (MEQ; [86]) and the ultra-short Munich Chronotype Questionnaire (µMCTQ; [87]), both administered at baseline and post-treatment.

## Brain imaging parameters

The outcomes of brain imaging will evolve in three types of brain parameter types: (1) structural parameters of gray matter, (2) structural parameters of white matter fiber bundles, and (3) functional brain parameters of the brain at rest:

1. With respect to the *structural parameters of gray matter*, they will encompass four main outcomes: thickness (THK), volume (VOL), surface area (SA), and gyrification (local gyrification index; LGI) across the entire brain.
2. Within the *structural parameters of white matter fiber bundles*, we will evaluate the integrity of major white matter fiber tracts by measuring as a main outcome the fractional anisotropy (FA) and mean diffusivity (MD), aiming to elucidate the impact of BLT on these structural brain properties.
3. Lastly, we will also evaluate the functional connectivity maps of specific brain networks (e.g., DMN, FPN, SN, and SMN) which are known to be impaired in depression. These functional connectivity maps are based on the blood-oxygenation level dependent (BOLD) signal under resting state conditions (resting state functional MRI).

Additionally, the pre- post-BLT assessment of these functional and structural brain networks will allow us to evaluate if the known functional and structural alterations of the brain in patients with depression changed after the BLT intervention. To evaluate the link between these biological markers and clinical symptom improvement, we will also look at the association between the earlier mentioned functional and structural brain changes and the change in depressive symptoms from pre- to post-BLT. This comprehensive and multiparametric (function and structure) MRI approach enables us to discern both structural and functional changes in the brain associated with the therapeutic effects of BLT.

## Light-related parameters

Personal light exposure will be evaluated using sensors developed by the Eindhoven University of Technology [88]. The light-related parameters for analysis may include logarithmic transformations of light level in lux illuminance (logEV) or melanopic equivalent daylight illuminance (mEDI) during specific periods, such as 1 h before EMA notifications, 3 h after waking, 3 h before bedtime, the interval between bedtime and wake time, and across the entire day. Time spent above certain light intensity thresholds (TaT), and the timing of these thresholds (mLiT) will also be examined. Key thresholds may include 5 lx at night, 1000 lx in the morning, and 250, 500, or 1000 lx during the day, as well as 100 lx in the evening. These parameters are flexible and can be adjusted as new research emerges. Light sensitivity will be estimated from the melatonin results, represented as the percentage decrease in melatonin production at specific time points during light exposure compared to the dim light condition, otherwise known as the light-induced melatonin suppression.

### Participant timeline {13}

**Fig. 2:**
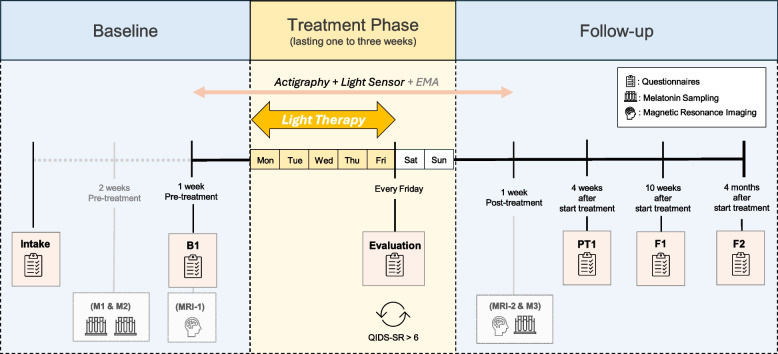
This figure presents a schematic overview of the study design. The study is divided into three phases: Baseline, Treatment, and Follow-up. Participants receive Bright Light Therapy from Monday to Friday, with the option to repeat the treatment up to three times if the score on the Quick Inventory of Depressive Symptomatology – Self-report (QIDS-SR; [70]) remains higher than 6. Gray-colored areas in the figure represent optional parts of the study protocol. EMA; Ecological Momentary Assessment

The study unfolds in three distinct phases: the Baseline Phase, the Treatment Phase, and the Follow-up Phase. The following sections detail the specific procedures for each phase of the study. A schematic overview of the study procedures can be found in Fig. 2.

### Baseline phase

In the Baseline Phase, participants undergo 1 to 2 weeks of measurements to assess their pre-treatment condition. The study begins with a 90-min face-to-face intake appointment (Intake). During this session, participants undergo the M.I.N.I. interview and complete the intake questionnaires. They also receive guidance on the proper use of the sensors and an explanation of how to use the BLT lamp. At the conclusion of the Intake, participants are randomized into one of the three treatment conditions.

One week before the start of therapy—typically on the following Monday—baseline examinations (B1) are conducted online. This includes completing the online questionnaires for B1 and a MADRS assessment via video call. Additionally, participants start with the EMA protocol and begin wearing the actigraphy watch and illuminance sensors. Those undergoing MRI assessments will also complete their baseline MRI scan (MRI-1) in the week before therapy. For participants involved in the melatonin assessments, an additional baseline week is added 2 weeks prior to the start of the treatment. This additional week includes the baseline DLMO assessment (M1) and an evaluation of light sensitivity (M2).

### Treatment phase

During the treatment phase, participants receive 1, 2, or 3 weeks of BLT within their assigned condition. Every Friday, depressive symptoms are assessed using the QIDS-SR, regardless of whether the participant received treatment that week. Treatment will be continued as long as QIDS-SR-score is higher than 6, with a maximum of 3 weeks. Treatment tolerability and mania symptoms are assessed using the YMRS after each week of BLT. EMA, actigraphy, and light logger usage continue throughout the treatment.

### Follow-up phase

After the treatment has been completed, EMA, actigraphy, and light logger usage are extended for an additional week and are discontinued on the Sunday at the end of this additional week. The post-treatment DLMO assessment (M3) and MRI scan (MRI-2) also occur within this first week after BLT.

Following these assessments, a post-treatment evaluation (PT1) is scheduled 4 weeks after the start of treatment, which includes a series of online questionnaires and an online MADRS assessment. During this meeting, participants receive general insights into their EMA data. The online follow-up questionnaires (F1) are sent 10 weeks after the start of treatment, with the second set of follow-up questionnaires (F2) distributed 4 months after the start of treatment, after which study participation is discontinued.

### Sample size {14}

#### Justification for the complete sample size

The sample size for this study was determined based on the power needed to answer our primary objective. A power analysis was initially conducted using G*Power 3.1 [89] to evaluate a simplified statistical approach comparing the MADRS scores across three treatment conditions using repeated measures ANOVA. This analysis was based on a clinically relevant effect size of *f* = 0.25, a significance level (α) of 0.05, and a desired power (1 − β) of 0.90, assuming a non-sphericity correction of 1. The results indicated that a total sample size of 210 participants would be necessary to detect significant differences.

To further validate this sample size, a simulated dataset was constructed using the R package *simr*, comprising 210 participants, each assigned to one of the three treatment conditions. The initial depression scores were set to a mean of 23.4 (*SD* = 13.2) based on research by Müller et al. [90]. Effect sizes of the treatment conditions were set to − 0.25, − 0.33, and − 0.4 corresponding to *Light@Home, LightCafé* and *LightCafé* + respectively, reflecting low to medium effect sizes. A power simulation of a linear mixed model using depression scores as the dependent variable, treatment condition and time point as fixed effects (including their interaction), and a random intercept per participant, revealed that the model achieved a high power of 98.30% (95% CI 97.29, 99.01) to detect significant interactions between time points and treatment conditions at an alpha level of 0.05, based on 1000 simulations. Assuming a drop-out rate of approximately 10%, we aim to include 231 participants in our trial.

### Justification for the EMA sample size

As the EMA is the most burdensome aspect of our study protocol, we carefully evaluated the feasibility of utilizing a subsample of the study population for this component. The primary objective of the EMA is to investigate the cross-lagged effects between sleep parameters and affect during therapy using a Random Intercept Cross-Lagged Panel Model (RI-CLPM). To determine the appropriate sample size for our analysis, we conducted a power analysis using the powRICLPM package in R, following the procedures outlined by Mulder (2023) [91]. The parameters for our analysis included 14 time points, an intraclass correlation coefficient (ICC) of 0.3, a correlation between random intercepts (RI_cor) of 0.3, a within-group correlation of 0.5, and expected effect sizes indicated by a correlation matrix with a moderate effect size of 0.4 for sleep and affect, as well as cross-lagged effects of 0.3. In our power analysis, we examined a sample size range from 100 to 231 participants, incrementing by 10, while employing 1000 Monte Carlo replications to ensure robust power estimates. The results indicated that a sample size of 120 participants would provide a power level of over 90%. As a result, we opted to include only 120 participants in the EMA component of the study. Following the data analysis, we will conduct a post hoc power analysis to evaluate the power of the RI-CLPM. If this analysis reveals insufficient power, we have the option to revert to a mixed model approach, which necessitates fewer participants to achieve similar power levels.

### Justification for the saliva sampling sample size

Saliva sampling is optional in the study protocol to reduce participation barriers. The main aim of the saliva sampling is to compare possible shifts in DLMO between conditions. A simulated dataset was created using the *simr* package in R, with a mean DLMO set at 23:00 and extensive between-person variance (*SD* = 120 min) and within-person variance (*SD* = 45 min). Using a mixed-effects model with a random intercept for participants, we simulated a sample of 72 participants measured at two timepoints (pre- and post-treatment). The analysis yielded a power of 96.40% (95% CI 95.05, 97.47) to detect a phase shift of 30 min in DLMO with an alpha level of 0.05. This high power suggests that even with the optional saliva sampling and a sample size of 72 we can confidently assess the effect of BLT on circadian rhythm changes.

### Justification for the MRI sample size

For the MRI assessments, our study will focus on a cohort of 60 participants recruited from LUBEC, as GGzE does not have the means to conduct MRI assessments. The main analysis of the brain data will involve a pre- vs. post-BLT comparison, without any comparisons among the three conditions (i.e., no between-subjects effect). A simple sensitivity power analysis, conducted using G*Power software for the intended statistical tests on the neuroimaging data (paired *t*-test), suggests that with a sample size of *N* = 60, we can achieve a power of 0.80 at an alpha level of 0.05, anticipating a small to medium effect size (Cohen’s *d* = 0.33), which aligns with findings from recent neuroimaging studies [59, 64, 66, 67].

Similar to the explorative subgroup analysis of the neuroimaging data comparing remitters and non-remitters will be based on past neuroimaging studies with a similar setting and having a sample size of minimal 20 participants per group [59]. This sample size is in adherence with recent publications in the field and accounts for the limitations encountered in a priori power size calculations of neuroimaging data [92, 93].

### Recruitment {15}

We intend to include participants year-round for approximately 2 years. The distribution of the recruitment burden is shared between two mental health clinics: GGzE’s aim is to include 171 participants, while LUBEC aims for 60. At GGzE, all individuals referred for BLT are approached for potential participation. Previous light therapy studies conducted at this center with the same enrolment strategy have achieved inclusion rates of approximately 100 participants per year. Given that this study is considered more extensive than previous research, we anticipate it will take approximately 2 years to reach the targeted enrolment of 171 participants. At LUBEC, BLT is offered for the first time, and only in conjunction with study participation. Recruitment efforts involve the collaboration with primary care and secondary mental health institutions for the referral of patients.

The rate of participant accrual is reviewed every half year. The number of participants who consented but did not qualify and the number of qualified, enrolled participants who dropped out will also be examined to detect patterns of insufficient recruitment rates. These will be addressed with modified recruitment strategies so that we can meet our recruitment goals.

## Assignment of interventions: allocation

### Sequence generation {16a}

Eligible participants are randomized using block randomization via the Research Manager data platform [94]. To ensure a balanced distribution among treatment groups and minimize potential biases, participants are stratified based on several key factors, including chronotype (categorized as early, intermediate, or late based on the MEQ [86], clinical diagnosis (differentiating between unipolar and bipolar conditions according to the M.I.N.I. assessment), and participation in the melatonin assessment (indicated as either yes or no).

### Concealment mechanism {16b}

A randomization table was generated using ResearchManager before the start of the trial. Access to the randomization table is restricted for the whole research team and can only be accessed by an independent researcher of the Eindhoven University of Technology not affiliated with the study. The randomization process in ResearchManager employs permuted block randomization with blocks of six to maintain balanced allocation across study groups.

### Implementation {16c}

All study participants are entered into ResearchManager during intake, and upon confirmation of eligibility and completion of intake questionnaires, they are randomized using the program’s algorithms. This process can be conducted by any member of the study team, including principal investigators, researchers, or study interns. During enrollment, researchers simply click a randomization button within ResearchManager, which then automatically assigns the participant to a study arm according to the randomization table.

## Assignment of interventions: blinding

### Who will be blinded {17a}

Participants, researchers, and care providers are informed about the allocated interventions. However, to maintain impartiality in evaluating depressive symptoms, the primary outcome assessors are kept blind to both the treatment arm and the assessment time. These assessors have no other role in the study and, therefore, will remain unaware of the study’s design and possible interventions.

### Procedure for unblinding if needed {17b}

N/a—all parties that could benefit from unblinding are already unblinded.

## Data collection and management

### Plans for assessment and collection of outcomes {18a}

#### Questionnaires

Several questionnaires are sent to the participant via ResearchManager at various timepoints in the study. An overview of these questionnaires, including the constructs they measure and the corresponding time points, is provided in Table 1. Each of these instruments has demonstrated well-established reliability and validity, ensuring their effectiveness in accurately measuring the intended constructs. To maintain high data quality, our research staff undergoes specialized training by experts, focusing on the consistent administration of the MADRS, M.I.N.I., and YMRS. Furthermore, we routinely assess inter-rater reliability for each MADRS administrator to ensure the accuracy and consistency of the assessments.

The following questionnaires and rating scales will be used during the study:

- **Mini-International Neuropsychiatric Interview (M.I.N.I.) PLUS 5.0** [69]: The M.I.N.I. is a brief, structured diagnostic interview designed to generate DSM-5 psychiatric diagnoses in approximately 30 min. It covers mood disorders (bipolar I & II, major depression, suicidality), anxiety disorders (social anxiety, panic, agoraphobia, generalized anxiety, PTSD, OCD), ADHD, eating disorders (anorexia, bulimia), substance use disorders (alcohol and other substances), and psychotic disorders. Its modular format allows rapid, reliable case‐finding in clinical trials and epidemiological studies. Test–retest reliability coefficients for major depression and anxiety diagnoses typically exceed *κ* = 0.70, and it shows good concordance with longer interviews such as The Structured Clinical Interview for DSM-5 [95]
- **Montgomery–Åsberg Depression Rating Scale (MADRS)** [78]: The MADRS is a clinician‐administered 10-item scale that quantifies depressive symptom severity over the preceding week. Each item (e.g., apparent sadness, inner tension, sleep, and appetite disturbances) is rated 0–6, yielding a total score from 0 to 60. The MADRS exhibits excellent inter‐rater reliability (ICC > 0.90) and sensitivity to treatment-induced change, making it a standard in antidepressant trials.
- **Quick Inventory of Depressive Symptomatology–Self-Report (QIDS-SR)** [70]: The QIDS-SR comprises 16 items mapping the nine core DSM-5 depressive domains (sad mood, anhedonia, concentration, self‐view, suicidal ideation, energy, sleep, appetite/weight, psychomotor changes) over the past 7 days. Responses use a 0–3 Likert scale, with composite scoring algorithms yielding a 0–27 total. Internal consistency is high (Cronbach’s *α* = 0.86–0.89), and the QIDS-SR correlates strongly with both the MADRS and HAM-D (*r* > 0.80), supporting its validity as a brief self‐report measure of depression severity.
- **Morningness–Eveningness Questionnaire (MEQ)** [86]: This 19-item self-report evaluates an individual’s circadian preference—“chronotype”—by asking about preferred timing for sleep, wakefulness, and peak performance. Scores classify respondents from “definite evening type” to “definite morning type.” The MEQ demonstrates good internal consistency (*α* ≈ 0.80) and has been validated against objective markers such as dim‐light melatonin onset.
- **Ultra-Short Munich Chronotype Questionnaire (μMCTQ)** [87]: The μMCTQ estimates chronotype via six items: usual sleep onset andsimi of work versus free days. By calculating the midpoint of sleep on free days corrected for accumulated sleep debt, it yields an estimate of intrinsic circadian phase. Test–retest reliability for sleep timing variables exceeds *r* = 0.90, and μMCTQ midpoints correlate well with full MCTQ scores.
- **Pittsburgh Sleep Quality Index (PSQI)** [84]: The PSQI is a 19-item self-report assessing sleep quality and disturbances over the past month across seven domains: subjective sleep quality, latency, duration, habitual efficiency, disturbances, medication use, and daytime dysfunction. Global scores > 5 indicate poor sleep. The PSQI’s internal consistency is acceptable (*α* = 0.75–0.83), and it discriminates insomniacs from healthy controls with a sensitivity of 89.6% and specificity of 86.5%.
- **Insomnia Severity Index (ISI)** [85]: The ISI consists of seven items rated 0–4 that probe the severity, impact, and perceived distress of insomnia symptoms. Total scores range from 0 to 28, with higher scores reflecting more severe insomnia. Internal consistency is excellent (*α* = 0.90), and the ISI correlates strongly with sleep diary metrics and polysomnographic indices, validating its use as a brief screening and outcome measure.
- **Consensus Sleep Diary(CSD)** [77]: A standardized daily log, the Consensus Sleep Diary captures subjective sleep–wake parameters—sleep onset latency, wake after sleep onset, total sleep time, time in bed, number and duration of awakenings, and morning sleep quality/refreshed ratings. It is the recommended unifying sleep diary for research, showing excellent agreement (*r* > 0.80) with actigraphy and flexibility for both EMA and weekly retrospective summary at follow‐up visits [77].
- **Short Form Health Survey (SF-12)** [82]: Derived from the SF-36, the SF-12 yields two summary scores—Physical Component Summary (PCS) and Mental Component Summary (MCS)—from 12 items covering domains such as physical functioning, role limitations, bodily pain, vitality, social functioning, and mental health. Despite its brevity, the SF-12 explains over 90% of the variance in SF-36 summary scores, exhibiting high test–retest reliability (ICC > 0.70) and construct validity across diverse populations.
- **Mental Health Quality of Life (MHQoL)** [83]: The MHQoL is a seven-dimension, four-level descriptive system (MHQoL-7D) plus a visual analogue scale (MHQoL-VAS, 0–10) for overall psychological well-being. Dimensions include self-image, independence, mood, relationships, daily activities, physical health, and outlook. It demonstrates good feasibility, content validity for mental health populations, and sensitivity to clinical change.
- **Credibility/Expectancy Questionnaire (CEQ)** [71]: The CEQ measures perceived treatment credibility and expectancy via six items: three assess credibility on a 1–9 scale and three assess expectancy on a 0–100% scale. Internal consistency ranges from *α* = 0.79 to 0.90, and early expectancy ratings predict clinical outcomes across psychotherapy and pharmacotherapy trials, underscoring its utility as a prognostic indicator.
- **Young Mania Rating Scale (YMRS)**: The YMRS is an 11-item clinician interview that quantifies manic symptoms—elevated mood, sleep, irritability, speech, thought content, motor activity, and insight. Items are weighted according to severity and duration (total score 0–60). Inter‐rater reliability is excellent (ICC > 0.85), and sensitivity to mood changes renders the YMRS a benchmark for bipolar disorder trials [74].
- **Adverse Event Scale for Light Therapy***:* A custom checklist will record the presence, frequency, and severity of known side effects of bright light exposure (e.g., headache, eyestrain, nausea), using Likert‐type ratings to facilitate systematic monitoring of tolerability throughout the intervention period.

**Table 1:** Overview of the questionnaires administered at different stages of the study

			***Treatment***			
	*Intake*	*B1*	*Evaluation*	*PT1*	*F1*	*F2*
**Mini International Neuropsychiatric Interview (M.I.N.I.)** [69] ***(clinical diagnosis of psychiatric disorders)***	X					
**Sociodemographics**	X					
**Current Medications and Therapies **	X			X	X	X
**Quick Inventory of Depressive Symptomatology Self-Report (QIDS-SR)** [70] ***(Self-reported Severity of Depressive Symptoms)***	X	X	X	X	X	X
**Montgomery-Åsberg Depression Rating Scale (MADRS)** [78] ***(Clinician-rated Severity of Depressive Symptoms)***		X		X		
**Morningness-Eveningness Questionnaire (MEQ)** [86] ***(Chronotype – Morningness/Eveningness preference)***	X				X	
**Ultra Short Munich Chronotype Questionnaire (µMCTQ)** [87] ***(Chronotype – Sleep Timing)***	X	X		X	X	X
**Pittsburgh Sleep Quality Index (PSQI)** [84] ***(Subjective Sleep Quality)***		X		X	X	X
**Insomnia Severity Index (ISI)** [85] ***(Severity of Insomnia Symptoms)***		X		X	X	X
**Short Form Health Survey - 12 items (SF-12)** [82] ***(Health-related functional outcomes)***		X		X	X	X
**Mental Health Quality of Life (MHQoL)** [83] (***Quality of Life related to Mental Health***)		X		X	X	X
**Credibility/Expectancy Questionnaire (CEQ)** [71] ***(Treatment credibility and participant expectations)***		X				
**Adverse Event Scale** ***(Occurrence and severity of adverse events)***			X			
**Young Mania Rating Scale (YMRS)** [96] ***(Symptoms of (hypo)mania)***			X			

This table gives an overview of the questionnaires utilized at various assessment points in the Baseline, Treatment, and Follow-up. Each questionnaire’s purpose is indicated alongside the time points at which it was administered indicated with an X. No questionnaires are administered during melatonin assessments (M1, M2, M3) or MRI sessions (MRI-1, MRI-2)

### Actigraphy

To assess sleep patterns and circadian rhythm parameters, participants wear a medical grade actigraphy watch, the MotionWatch 8 (CamNtech, Cambridge, UK), throughout the treatment period, baseline week, and post-treatment week. This validated medical device contains an internal accelerometer that reliably captures movement data in 30-s epochs. Participants receive clear instructions on how to wear the watch and are encouraged to keep it on during all the waking and sleeping hours to ensure accurate and continuous data collection.

### Personal Illuminance Sensor

Participants wear a small, box-shaped light sensor developed by the Eindhoven University of Technology [88] near their clavicle from waking to bedtime during the baseline, treatment, and follow-up weeks to assess their light-related behaviors. This device uses a TAOSTCS34725 optical sensor and an onboard EEPROM memory of 64 kb. The sampling window is set to 30 s, meaning the device takes a light measurement every 10 s and averages these for the time window. To ensure optimal data collection, participants are instructed to turn the sensor upside down at bedtime to prevent it from measuring light exposure that does not actually fall on the eyes. Additionally, participants are advised to remove the sensor in wet conditions, as it is not water-resistant.

### Ecological Momentary Assessment (EMA)

Momentary affect states, momentary energy levels, and subjective sleep parameters are assessed using an ecological momentary assessment (EMA) approach in a subsample of 120 participants. Throughout the baseline, treatment, and post-treatment weeks, participants receive eight EMA notifications daily via a mobile application called M-Path [97]. Each notification, which takes approximately 1 min to complete, occurs at semi-random intervals across eight 105-min periods throughout the day. Participants have the flexibility to choose their preferred notification schedule, receiving alerts either between 7:00 AM and 9:00 PM, 8:00 AM and 10:00 PM, or 9:00 AM and 11:00 PM. To increase the validity of the momentary states, notifications become inaccessible 30 min after they are sent. In addition to the eight momentary notifications, participants are asked to complete a sleep diary each morning to assess subjective sleep parameters, as well as an evening questionnaire assessing daytime sleep, caffeine and drug intake, and treatment adherence.

### Melatonin assessments

In a subset of participants (*n* = 72), salivary melatonin samples are collected in three additional evening sessions: two during the measurement week before baseline (M1 – DLMO and M2 – Light Sensitivity), and one in the week following treatment (M3—DLMO). Participants collect saliva samples at home by swabbing a cotton swab in their mouth and placing it in a salivette®. Samples will be promptly refrigerated and shipped to the lab within 3 days. In the lab, samples are centrifuged and stored at − 20 °C for analysis. Melatonin concentration in the saliva samples is analyzed in the lab of Chrono@Work B.V. (Groningen, The Netherlands) with a double-antibody radioimmunoassay (RIA) (Direct Saliva Melatonin kit, NovoLytiX GmbH, Switzerland). Specific saliva sampling procedures depend upon the type of assessment (DLMO or Light Sensitivity).

#### Dim Light Melatonin Onset (DLMO) assessment

DLMO is assessed at M1 and M3. Participants provide eight saliva samples on each collection day, starting 4 h before their habitual sleep onset time (determined from the sleep diary in the baseline) and ending 1 h after. The samples are collected at hourly intervals, with two extra collections 30 min before and after habitual sleep onset. To ensure a dim light environment, participants are instructed to keep their home lighting as dim as possible and reduce the intensity and contrast of computer and phone screens to a minimum. Participants are also asked to sit away from Lamps, maintain a distance of at least 3 m from the TV, and close curtains.

#### Light Sensitivity

To evaluate light sensitivity, we estimate the light-induced melatonin suppression during baseline (M2). Five salivary melatonin samples are collected by the participants at 30-min intervals, beginning 1 h before habitual sleep onset and concluding 1 h after. Participants remain in the same lighting conditions as in the DLMO assessment, but 30 min before their habitual sleep onset time they are exposed to 30 min of light with an illuminance of approximately 250 lx at eye level and at distance of approximately 70 cm from a modified BLT lamp.

### Brain imaging

Sixty participants at LUBEC undergo two brain imaging sessions at the 3 Tesla MRI facility of the Leiden Institute for Brain and Cognition (LIBC) of the Leiden University Medical Center (LUMC) in Leiden. The First MRI session takes place the week before BLT, and the second session is conducted the week after BLT completion. Each session has a maximum duration of 60 min (including preparation, positioning of participant, and scanning). The brain scans are performed on a Philips 3 T MRI system (Best, The Netherlands) at the Leiden Institute for Brain and Cognition (LIBC) of the Leiden University Medical Center (LUMC) in Leiden, The Netherlands. The following MRI imaging sequences are used in each session:

- *Structural High-Resolution T1*: This sequence captures detailed information about GM, including parameters like THK, VOL, SA, and LGI. It is also essential for subsequent functional scan analysis. For this, we will use a high-resolution T1 weighted image with the following parameters: repetition time (TR) = 7.9 ms, echo time (TE) = 3.5 ms, flip angle = 8°, field of view (FOV) = 250 × 195.83 × 170.5 mm, 155 slices.
- *Diffusion Weighted Imaging (DWI)*: This sequence enables the assessment of the brain’s structural connectivity by examining the primary WM tracts. Data from the DWI is analyzed using a diffusion tensor imaging technique (DTI). For this, we use a DTI high-iso sense sequence with the following parameters: TR = 6500 ms, flip angle = 90°, FOV = 224 × 224 × 125.4 mm, number of slices = 57, shim size = 100 × 100 × 100. SENSE = 2.5. Diffusion-sanitizing gradient encoding is applied in 32 directions with a diffusion weighted factor of b = 1000 s/mm^2^ and two b0 (b = 0) images. Images are acquired parallel to the anterior–posterior commissure.
- *Functional Resting State*: This sequence provides information about the functional connectivity of brain networks while at rest. For this, a T2*-weighted using gradient-echo planar imaging (EPI) sequence is used. The parameters entail: TR = 2200 ms, TE = 30 ms, flip angle = 80°, number of slices = 40 transverse, slices thickness = 2.75 mm, FOV = 22 × 220 × 120.725.
- *Structural T2w Image*: To better reconstruct the structural images and obtain exact values of THK, VOL, SA, and LGI within the analysis program FreeSurfer (http://surfer.nmr.mgh.harvard.edu/), we also perform a structural T2-weighted 3D Flair Image at the end of the scan with the following parameters: TR = 4800 ms; FOV = 250 × 250 × 182,56; 326 slices.

### Plans to promote participant retention and complete follow-up {18b}

An interactive feedback session is planned with the participants during the post-treatment assessment where we evaluate their individual study outcomes, focusing mainly on the EMA data. This offers a personalized incentive for continued participation. Online questionnaires and automated reminders are integrated to ensure data retention. Additionally, personal contact with participants is maintained throughout the study to promote adherence to the protocol.

### Data management {19}

ResearchManager is utilized for data entry and initial storage of all information gathered through questionnaires. Study personnel will complete the study forms for each appointment within the electronic Case Report Form (eCRF), while self-rated questionnaires will be dispatched to participants via email through the platform. In instances where questionnaires are completed on paper, the source documents will be securely stored in a locked filing cabinet with restricted access limited to research personnel. Prior to locking the data into the system, all information will undergo validation checks by either EV, OGRO, or NA. Data collected through ResearchManager will be securely stored in encrypted format on a protected server. Following the conclusion of data collection, it can be exported to Excel sheets, which will then be stored on the local servers of both Eindhoven University of Technology and Leiden University.

EMA data will be gathered using the M-Path application [97] and stored in encrypted format on M-Path’s secure server, which complies with Dutch NEN 7510 and international ISO 27001 standards for information security management. Subsequent to the final data collection, data is exported to Excel sheets and stored on local research servers. Saliva collected for melatonin assessments will be dispatched to Chrono@Work B.V. via post by the participants themselves. Melatonin concentrations will be determined in the laboratory, and the saliva sample will then be discarded safely. Data will be shared in Excel sheets via secure email and stored on local research servers. Data collected via actigraphy, or personal light exposure sensors will be transformed into Excel sheets using the Motionware program or the LightlogControl program, respectively. These sheets will also be stored on local servers. MRI data will be acquired at the university hospital and will be stored on the protected local server of Leiden University. All study-related data will be archived for a minimum duration of 15 years.

### Confidentiality {27}

Information collected about participants during this clinical investigation is treated confidentially. The collection and processing of participants’ personal information is limited to what is necessary to ensure the study’s scientific practicability, the evaluation of efficacy, adherence, side effects, and the investigational product’s safety. All data is pseudo-anonymized using participant identification numbers.

### Plans for collection, laboratory evaluation and storage of biological specimens for genetic or molecular analysis in this trial/future use {33}

Saliva will be collected from the participants that consent to the melatonin assessments. Melatonin concentration in saliva will be analyzed in the lab of Chrono@Work B.V. (Groningen, The Netherlands) and destroyed afterwards. Saliva cannot be used in future studies.

## Statistical methods

### Statistical methods for primary and secondary outcomes {20a}

This analysis plan provides the initial framework for examining primary and secondary outcomes. A significance level of 0.05 will be applied unless otherwise specified, with corrections for multiple comparisons (e.g., Bonferroni) used when necessary. All analyses will follow the intention-to-treat principle, accounting for the nested structure within centers if appropriate. The analyses outlined here may be subject to minor modifications based on advancements in the field. Should any deviations from the original plan occur, these will be clearly documented and justified. Our reporting will adhere to the guidelines set by the CONSORT statement [98].

### Study Sample Description

We will report the descriptive statistics of socio-demographic and clinical variables for the whole sample, per site, and per treatment arm. In specific analyses involving subsamples (e.g., melatonin assessments, EMA, and MRI), we will provide socio-demographic and clinical characteristics of the subsample and report any distinctions from the overall sample*.*

### Primary Study Outcome – Clinical Improvement between Treatment Conditions

To compare the three different BLT administration strategies in terms of clinical improvement (measured as the difference in MADRS scores between baseline and post-treatment assessments), we will employ a multilevel mixed-effects modelling approach. MADRS scores will serve as the dependent variable, with a fixed effects for timepoint (*pre- and post-treatment*), and the interaction between timepoint and treatment arm (*Light@Home, LightCafé*, and* LightCafé* +). Random intercepts for participants and study centers will be included to account for individual differences and center-level variability, but only if they improve model fit, as determined by evaluation of the Akaike Information Criterion (AIC) and Bayesian Information Criterion (BIC). Random slopes for time within participants will also be considered and included based on the same criteria. Socio-demographic (age, sex) will be evaluated for potential confounding effects, similar to the credibility and expectancy of the treatment arms as assessed with the CEQ. The primary analysis will focus on the interaction between timepoint and treatment arm to determine whether clinical improvement differs across the three BLT strategies. Furthermore, multilevel logistic regression will be used to test differences in response (yes/no) and remission (yes/no) between treatment arms using the same structure and procedure as above.

### Secondary Study Outcomes

#### Subjective Assessment of Depressive Symptoms, Relapse Rates and Functional Outcomes

Multilevel mixed-effects models will be constructed to assess depressive symptom improvement, measured weekly during therapy and at follow-up assessments using the QIDS-SR scores as the dependent variable. Fixed effects will include timepoint as an categorical variable (baseline, 1 week, 2 weeks, 3 weeks, 4 weeks, 10 weeks, and 4 months), and the interaction between timepoint and treatment condition (*Light@Home*,* LightCafé*, and *LightCafé* +). Random intercepts and slopes will be included for participants and study centers, as well as random slopes for time within participants, based on model fit criteria. Confounding variables (age, sex, clinical diagnosis, and credibility/expectancy of treatment) will also be evaluated and included as necessary. A variable will be added to account for treatment duration (1 week, 2 weeks, 3 weeks). Similar models will be considered for analyses of relapse rates, functional outcome, and Quality of Life with inclusion of the respective timepoints as fixed effects (see Table 1).

#### Time to remissionand time to response analysis

To assess differences in time to remission, we will utilize survival analysis techniques. Participants will be categorized based on the number of weeks until remission (1 week, 2 weeks, 3 weeks, or censored if not reached within the treatment duration). Kaplan–Meier survival analysis will be employed to visualize the probability of achieving remission over time between treatment conditions (*Light@Home*,* LightCafé*, and *LightCafé* +). Additionally, we will apply Cox proportional hazards modelling to evaluate the effects of treatment condition and covariates (age, gender, clinical diagnosis) on the time to remission (outcome). Results will be presented with survival curves and hazard ratios. Similar analyses will be conducted on time to response.

#### Treatment effects on sleep parameters

To analyze changes in sleep patterns over time and between treatment conditions, multilevel mixed-effects models will also be utilized. Sleep outcomes include the following outcomes from both sleep diaries and actigraphy: sleep duration, sleep onset latency, wake after sleep onset, midsleep timing, sleep onset, and sleep offset. Additionally, sleep quality will be calculated from sleep diary and sleep fragmentation from actigraphy. The fixed effects structure will encompass time (*days since treatment start*), and the interaction between time and treatment condition (*Light@Home*,* LightCafé*, and* LightCafé* +), allowing us to explore how sleep patterns evolve throughout the intervention and whether these changes differ across conditions. The inclusion of random intercepts for participants and study centers, along with random slopes for time within participants, will be evaluated based on model fit criteria. Additionally, the model will incorporate a factor indicating whether a day is a weekend or weekday to account for social jet lag experienced by participants.

Furthermore, we will investigate the relationship between subjective sleep measures from sleep diaries and objective data obtained from actigraphy through correlation analyses. Next, we will assess the total PSQI and ISI scores over time and between conditions using a linear mixed model approach as described above, focusing on the timepoints of *Baseline, 4 weeks post-treatment, 10 weeks post-treatment*, and *4 months post-treatment* instead of *days since treatment start.*

#### Circadian effects of BLT and their relation to the antidepressant outcomes

To analyze changes in circadian parameters, including DLMO, Phase Angle Difference, inter-daily stability, and intra-daily variability, multilevel mixed-effects models will be employed. The fixed effect’s structure will incorporate time (*Baseline vs. Post-treatment*), and the interaction between time and treatment condition (*Light@Home*,* LightCafé*, and *LightCafé* +). Inclusion of random intercepts for participants and study site and random slopes for time within participants will be evaluated based on model fit criteria, including AIC and BIC. Circadian effects will be compared between timepoints by evaluating the fixed effect of time in the model, while the effect of the treatment condition will be interpreted by assessment of the interaction term between time and treatment condition.

Following the assessment of changes in circadian parameters (e.g., DLMO, Phase Angle Difference, inter-daily stability, and intra-daily variability), multilevel mediation analyses will be conducted to determine whether these changes mediate the relationship between timepoint and depressive symptoms (QIDS-SR). Each mediator will be tested individually using an approach based on Bauer et al. [99] which includes decomposing within- and between-person components to yield valid indirect effect estimates in nested data. We will apply bootstrapping (5000 samples) to estimate the indirect path (ab) and construct 95% confidence intervals for each mediator. In addition, moderation analyses will examine whether baseline depression severity or sleep quality interact with treatment conditions to affect depressive outcomes. Results will report direct effects (c′), indirect effects (ab), and any interaction effects (a × b), along with the corresponding confidence intervals and *p*-values.

#### Dynamics of sleep, affect, and energy levels throughout therapy

The primary objective of the EMA is to investigate the dynamic interplay between sleep, affect, and energy levels during BLT. We aim to uncover whether the antidepressant effects of BLT primarily result from improvements in mood, sleep, daytime energy levels, or a combination of these factors. For statistical analysis, we will first calculate daily averages for all momentary measures. If fewer than three measures are available for a given day, the daily average will be set to not applicable (*NA)*. To isolate within-person variability, all measures will subsequently be person-mean-centered. Additionally, baseline measures—such as depressive symptoms and sleep quality—will be grand-mean centered.

To analyze the data, we will specify a Random Intercept Crossed-Lagged Panel Model (RI-CLPM) that focuses on the relationships between sleep parameters, affect, and energy levels over time. This model will incorporate dynamic variables, specifically the person-mean centered scores for sleep parameters, affect, and energy levels measured at the day level, alongside trait variables represented by grand-mean centered scores from baseline assessments, including severity of depressive symptoms and sleep quality. The model structure will include random intercepts for each participant, autoregressive paths to assess the stability of each variable, and cross-lagged paths to evaluate how one variable at a previous time point influences another variable at a subsequent time point. To estimate the RI-CLPM, we will utilize the *lavaan* package in R, employing maximum likelihood estimation for model fitting.

Prior to model construction, we will conduct an exploratory analysis to identify the most relevant parameters related to sleep, affect, and energy levels for inclusion in the model. This step is crucial to ensure that we accurately measure the constructs involved and capture the dynamics effectively. The exploratory analysis will involve visualizations, such as time series plots, lag plots, and scatter plots, to assess relationships and patterns in the data. Additionally, we will investigate how the variables change over time, seeking patterns in daily fluctuations and overall trends throughout the therapy period. Correlations between different variables at various time scales will also be examined to identify initial relationships that could inform model selection.

Based on the findings from the exploratory analysis, we will construct a model that best represents the dynamics of sleep, affect, and energy levels. We may incorporate covariates and trait-level variables, such as demographic factors, baseline depressive symptoms, and sleep quality, which could influence the relationships being studied. In our reporting, we will present the overall model fit indices (e.g., comparative fit index, Tucker–Lewis index, root mean square error of approximation) to assess the adequacy of the RI-CLPM. We will detail the autoregressive paths indicating the stability of each variable over time, as well as the cross-lagged paths, which reveal how changes in one variable at a previous time point influence changes in another variable at a subsequent time point. This systematic examination aims to enhance our understanding of the therapeutic mechanisms underlying BLT and how improvements in sleep, affect, and energy levels interact throughout the treatment process.

Following the data analysis, we will conduct a post hoc power analysis to evaluate the power of the RI-CLPM. If this analysis reveals insufficient power, we have the option to revert to a mixed model approach, which necessitates fewer participants to achieve similar power levels.

#### Identification of predictors of response and remission

Another aim of the study is to identify combinations of patient characteristics and behaviors that predict treatment response and remission. To achieve this, we will conduct AIC penalized bootstrapped stepwise logistic regression in both forward and backward directions, examining predictors of remission and treatment response in two separate models. The examined predictors will include:

- Demographic Factors: *Age, Sex, Educational Level*
- Clinical Characteristics: *Clinical Diagnosis, Severity of Depressive Symptoms, Diurnal Variation in Affect*
- Sleep-Related Factors: *Subjective Sleep Quality, Sleep Efficiency, Sleep Duration, Sleep Onset Latency, Midsleep Timing, Wake after Sleep Onset, Severity of Insomnia Symptoms, Energy Levels*
- Circadian Factors: *Circadian Phase, Intradaily Stability, Intradaily Variability, Phase Angle Difference between DLMO and Sleep Onset, chronotype.*
- Light-related parameters: *Light Sensitivity*
- Expectations: *Expectations of Treatment*

All predictors will be measured either at intake or during the baseline week. Prior to the stepwise regression analysis, we will assess multicollinearity by calculating the Pearson correlation coefficient and Variance Inflation Factor (VIF). Variables exhibiting a Pearson correlation coefficient greater than 0.8 or a VIF score exceeding 10 will be removed to mitigate multicollinearity and examined separately. A final competitive regression model will be run with all significant predictors.

To ensure the robustness of our models, we will run 1000 bootstrap samples and retain predictors that are significant in at least 60% of the bootstrapped samples. We will also calculate partial R-squared values for all predictors in the regression models to quantify the proportion of variance in the dependent variable explained by each specific independent variable while controlling for the influence of others. Finally, we will conduct variance partitioning to investigate the unique and shared variance explained by the predictors. By comparing the explained variance from regression models of individual predictors against those with combinations of predictors, we will compute both unique and shared variance. The vegan package in R will be used for variance partitioning, and results will be visualized using Euler plots from the Euler package.

### Neural Correlates of BLT

#### Preprocessing and data cleaning

Neuroimaging data (functional and structural) will be visually inspected and subjects with artifacts in the images (e.g., based on movement) will be discarded from the analysis. All brain data will be transformed into the brain imaging data structure (BIDS), in order to be shareable and easily accessible for further steps [100]. Data will then be pre-processed and analyzed using existing standardized neuroimaging pipelines depending on the data types (structural or functional). Functional data will be pre-processed using fMRIPrep [101, 102].

#### Neuroimaging data types and software

We will gather three types of data based on the outputs from the four scanning sequences employed.

The first type is structural imaging data, which includes measures of gray matter (GM), white matter (WM), and LGI. This data will be pre-processed using the standardized FreeSurfer analysis pipeline (http://surfer.nmr.mgh.harvard.edu/). The preprocessing steps are automated and briefly include motion correction, intensity normalization, skull striping, segmentation in GM and WM, and inflation of these surfaces. Afterwards the extraction of specific GM and WM metrics is performed, this encompasses, among others, THK, VOL, SA, and LGI.

The second type is WM tract data obtained from DTI sequence. We will focus on extracting properties of the main WM tracts, specifically FA and MD. For this, we will use a probabilistic tracking analysis approach implemented as a standardized analysis pipeline of Freesurfer, namely the TRActs Constrained by UnderLying Anatomy (TRACULA) [103, 104].

The third type is functional connectivity data based on the BOLD signal of the resting state functional scan; it will allow to assess the functional connectivity properties of certain brain networks under resting state conditions using standardized pipelines from the CONN toolbox [104].

#### Analysis of brain function and structure before vs. after BLT

Brain function and structure will be analyzed between pre- vs. post-BLT intervention in the whole sample of 60 participants at the Leiden study site.

For the structural parameters of GM, the longitudinal two-stage analysis framework of general linear modelling (GLM) will be used. This is implemented as an automated analysis pipeline in the longitudinal data analysis framework of FreeSurfer. In brief, this method first extracts the regional GM parameters (VOL, SA, THK, and LGI) at the level of each subject and each brain point (vertex) for each hemisphere separately. In a second step, a group level analysis will be performed by using a repeated measures ANOVA. Hereby, we will compare the structural parameters THK, VOL, SA, and LGI between pre- and post- BLT while including age, sex, and treatment arm (*Light@Home, LightCafé, LightCafé +*) as covariates and to correct for their potential confounding effects. To account for multiple testing across the whole brain, we will perform Monte Carlo simulations with 10,000 iterations to identify significant contiguous clusters of vertex-wise group differences (*p* < 0.05).

For the analysis of the structural WM properties of main WM tracts, the probabilistic tractography pipeline TRACULA will be used. This automatic pipeline is first pre-processing the DTI images (correcting for image distortions, eddy currents, and B0 field inhomogeneities). Afterwards, head motion measures are extracted which can be used in further statistical analysis as covariates, co-registration of DTI and anatomical images is performed, the registration of individual images to a common space occurs, then the probabilistic distributions of 42 major WM fiber tracts are reconstructed from which the main properties of WM bundles can be extracted (FA and MD). Then the pipeline enters the longitudinal analysis stream which involves as a main statistical approach, the earlier mentioned GLM model. Hence, a repeated measures analysis of variance (ANOVA) will be performed on group level in which the comparison of FA and MD between pre- and post-BLT will be evaluated. Afterwards, clusterwise correction for multiple comparisons using Monte Carlo simulations with 10,000 iterations will be employed to account for multiple testing effects.

For the analysis of brain function, the connectivity maps of main functional resting state networks will be analyzed using the CONN toolbox [104] from the software Statistical Parametric and Mapping (SPM). For this, we will replicate the study by [59] [59]. Hence, we will both investigate inter-network connectivity (in DMN, FPN, SMN, SN) but also at the intra-network connectivity as a post hoc test using a graph analysis. In the first level analysis, the network properties of each network in each subject will be established. In the second level analysis, a group-level analysis will be performed to analyze the differences in the brain networks (DMN, FPN, SMN, SN) between pre- and post-BLT. A significance threshold of 0.05 will be set and a false discovery rate (FDR) correction method will be applied to correct for multiple comparisons.

#### Analysis of functional and structural brain correlates and clinical symptoms

To assess whether the encountered brain changes are linked to the clinical symptom improvement, multiple regression analysis will be performed. Hence, brain measures (functional or structural) which showed changes between the pre- vs post-BLT intervention will be correlated with the clinical symptom change scores of depression (QIDS-SR, MADRS). More specifically, the brain measures involved are functional connectivity measures of specific brain networks (DMN, FPN, SMN, SN) which showed changes between pre and post BLT, the structural measures—GM properties (THK, VOL, SA) but also WM tracts properties (FA, MD) which showed changes between pre-post BLT.

Analysis of melatonin and BLT linked brain changes.

We will evaluate if a change in DLMO is also linked to the encountered brain changes on functional and structural level. Hence a multiple regression analysis will be performed between the encountered brain changes as predictors (in function and structure) and the DLMO changes as outcome measure in the analysis.

To evaluate the potential link between circadian light sensitivity and brain alterations in depression, a multiple regression analysis will be performed. Hence the melatonin suppression will be used as a predictor and functional and structural brain changes and as outcomes in the model.

### Interim analyses {21b}

N/a—we are not conducting any interim analyses.

### Methods for additional analyses (e.g., subgroup analyses) {20b}

Exploratory subgroup analyses will be conducted to examine variations in the effects of different administration strategies on depressive symptoms, sleep, and circadian rhythms across various depression diagnoses. Additionally, the whole brain structure and functional parameters will be compared between the three treatment arms. As a post hoc explorative analysis the functional and structural brain parameters will be compared between remitters vs non-remitters. Multiple comparison corrections for repeated measures will be done.

Furthermore, we will explore the relationship between treatment adherence (as measured by self-report and light sensor data) and treatment effectiveness. Adherence will be operationalized as the percentage of completed light therapy sessions (based on sensor data, the timing of light exposure relative to the prescribed schedule, and daily self-reported compliance). These adherence indicators will be added as covariates in regression models predicting changes in depressive symptoms, sleep, and circadian phase markers. Primary outcome models will also include medication type and dose as time-varying covariates to test for interaction with BLT. Exploratory subgroup comparisons (e.g., on versus off antidepressant) may further probe whether concurrent treatments alter BLT’s therapeutic impact.

### Methods in analysis to handle protocol non-adherence and any statistical methods to handle missing data {20c}

The data analysis is an intent-to-treat analysis with participants analyzed as randomized, regardless of adherence to the treatment protocol. Additionally, completer analyses will be conducted, including only those participants who have both pre-treatment and post-treatment data. Furthermore, per protocol analyses will focus on participants who completed at least 80% of the treatment (i.e., attending 4 out of 5 days of BLT per week). Any substantial differences between these analytical approaches will be evaluated and reported.

We will describe the extent and patterns of missing MADRS scores and covariates overall and by treatment arm. To evaluate whether missingness is completely at random (MCAR), we will apply Little’s MCAR test and examine associations between missingness and key variables (e.g., age, sex, diagnosis, depression severity, treatment expectancy). Under a plausible missing-at-random (MAR) assumption, we will perform multiple imputation by chained Eqs. (10 imputations) as a sensitivity analysis, refitting the primary multilevel mixed-effects models in each imputed dataset and pooling estimates via Rubin’s rules. We will then compare results from the complete-case analysis and the MAR sensitivity analysis to our main intent-to-treat (observed-data) findings. Any notable discrepancies in effect estimates, model fit, or inferential conclusions across these approaches will be reported.

### Plans to give access to the full protocol, participant-leveldata, and statistical code {31c}

Participant-level data (from consenting participants) and the corresponding statistical code will be made available upon request. Parts of the data, i.e., data published in peer-reviewed journals will be stored in the public repository. The aim of this step is that other researchers can have access to include these datasets in meta-analyses, reviews, or similar or enable replication of our study. This data is pseudonymized and will not contain any personal information. The complete data set will be stored on the local research institutional research servers in a pseudonymized fashion. We will adhere to the requirements of the DataverseNL platform for data archiving.

## Oversight and monitoring

### Composition of the coordinating center and trial steering committee {5d}

**Principal Investigators**

Members: Niki Antypa and Yvonne de Kort

Past Members: Luc Schlangen

Roles and Responsibilities:

- Overall study governance
- Annual update report to the National Ethical Review Board
- All (S)AE reporting to the Ethical Review Board
- Finance oversight, budget administration, contractual negotiations
- Provide scientific advice to Lead Investigators

Meetings: Weekly with lead investigators and monthly with the steering committee.

**Lead Investigators**

Members: Emma Visser and Oana Georgiana Rus-Oswald

Roles and Responsibilities:

- Site-level recruitment, informed consent and enrolment
- CRF completion, data query resolution and source-data verification
- Patient follow-up per protocol schedule
- Drafting and revising protocol sections, writing study progress reports
- Coordinating and hosting Steering Committee meetings

Meetings: Weekly with lead investigators and monthly with the steering committee.

**Steering Committee**

Current Members: A.J.W. van der Does, M.C.M. Gordijn, M.C. Marcelis, T. Seetsen, C.J.P. Simons

Past Members: P.P. Oomen.

Roles & Responsibilities:

- Final sign-off of protocol and any substantive amendments
- Ongoing review of study progress: recruitment, retention, data completeness
- Approve operational changes to facilitate trial conduct
- Resolve major operational or scientific disputes

Meetings: Monthly meetings with the principle investigators lead investigators and steering committee

### Composition of the data monitoring committee, its role and reporting structure {21a}

This study does not include a Data Monitoring Committee as BLT is a low-risk intervention and study procedures are straightforward and do not require ongoing oversight.

### Adverse event reporting and harms {22}

Adverse events (AE) are defined as any undesirable experience occurring to a subject during the study, whether or not considered related to BLT. All AE reported spontaneously by the subject or observed by the investigator, or their staff will be recorded. Additionally, a weekly screening of AE will be conducted. All AEs will be followed until they have abated, or until a stable situation has been reached. Depending on the event, follow-up may require additional tests or medical procedures as indicated, and/or referral to the general physician or a medical specialist.

### Frequency and plans for auditing trial conduct {23}

N/a—no auditing.

### Plans for communicating important protocol amendments to relevant parties (e.g., trial participants, ethical committees) {25}

A “substantial amendment” is defined as an amendment to the terms of the Medical Research Ethics Committee (MREC) application, or to the protocol or any other supporting documentation, that is likely to affect to a significant degree:

- The safety or physical or mental integrity of the subjects of the trial.
- The scientific value of the trial.
- The conduct or management of the trial; or.
- The quality or safety of any intervention used in the trial.

All substantial amendments will be submitted for approval to the MREC and to the competent authority. For non-substantial amendments, only a notification will be sent to the accredited MREC, which will be recorded and filed by the sponsor.

### Dissemination plans {31a}

The results of the study will be submitted for publication in international peer-reviewed journals adhering to applicable privacy laws and regulations. The main study findings will be communicated to study participants after data collection is complete and data have been analyzed.

## Discussion

In this paper, we present a protocol for a multicenter clinical trial aimed at addressing practical implementation challenges associated with BLT. Although decades of research have confirmed BLT’s antidepressant potential across a spectrum of mood disorders, three persistent questions still exist: How should we deliver BLT most effectively? Which patients benefit the most? And by what biological and behavioral pathways does light exert its mood‐lifting effects? This study therefore extends beyond a mere assessment of BLT’s efficacy, focusing on three key objectives: (1) comparing the effectiveness of three distinct BLT administration strategies, (2) identifying personal characteristics and behaviors that predict responses to BLT, and (3) investigating the chronobiological and neurobiological pathways that underpin BLT’s efficacy.

What sets this trial apart is its comprehensive effort to facilitate a multi-faceted approach to BLT by integrating contextual and personalized chronobiological factors in a transdiagnostic manner, including patients with several mood disorders. A central element in this is the innovative LightCafé. Unlike solitary, clinic-bound approaches, the LightCafé brings patients together in a welcoming, café-style space, where social interaction and group dynamics reinforce healthy routines and bolster adherence. Complemented by individualized coaching on sleep hygiene, nutrition, activity rhythms, as well as personalized chronotherapeutic interventions, the *LightCafé* transforms solitary light exposure into an immersive, routine-building experience. Such a multi-modal approach to depression treatment could lead to better and longer-lasting effects. By running sessions much like group therapy, the *LightCafé* can accommodate multiple patients simultaneously, thereby reducing waiting times, lowering per-patient costs (no need for individual light-box purchases), and cutting down on repeated clinic visits—relieving both families’ out-of-pocket expenses and health-system burdens. Its built-in social context also improves adherence and eases caregiver time commitments, while freeing up scarce therapeutic resources for more complex cases. While other similar environments might also yield effective outcomes, the primary goal here is to emphasize the value and necessity of a multifaceted therapeutic environment.

To benchmark the added value the *LightCafé* and the personalized chronotherapeutic interventions, we contrast the two administration strategies against an active home-based control (Light@Home). Opting for Light@Home as our control arm reflects a pragmatic choice: although it precludes attribution of observed effects solely to light exposure, it provides a realistic comparator that mirrors how many patients already receive BLT and is therefore a valuable comparator in our search for optimization of administration. It should however be noted that even though we adhere to Dutch BLT guidelines for depression, the field still lacks consensus on optimal BLT dosing—specifically wavelength, intensity, and overall treatment duration—so true optimized delivery of BLT still warrants further investigation.

Besides these pragmatic, real-life objectives, our study also aims to identify individual characteristics predicting responses to BLT, examining a range of socio-demographic, clinical, sleep-related, circadian, and light-related factors. We will employ advanced statistical techniques to uncover strong predictors of positive outcomes, enhancing clinical decision-making and paving the way for a more personalized approach to BLT. The trial also explores the mechanisms underlying BLT’s efficacy through mediation analysis, assessing whether and how changes in circadian rhythm mediate its antidepressant effects. Using EMA data analyzed via random-intercept cross-lagged panel modelling (RI-CLPM), we will capture real-time fluctuations in mood, sleep, and energy levels, clarifying whether BLT primarily targets mood, sleep, energy, or a combination of these factors. Neurobiological analyses via MRI will further elucidate structural and functional brain changes associated with BLT, including assessments of connectivity and activity patterns.

In summary, our study represents a significant advancement in the optimization of BLT for clinical practice. By employing innovative methodologies and conducting comprehensive assessments, we aspire to inform the development of clinical guidelines that enhance the accessibility and efficacy of BLT in real-world settings. This research not only seeks to improve treatment outcomes for individuals with mood disorders but also paves the way for future investigations into personalized therapeutic approaches in mental health care. The innovative *LightCafé* model enables the integration of personal characteristics and advanced methodologies and constitutes a robust framework for optimizing BLT and its real-world applicability, with the potential to substantially impact clinical practice and improve patient outcomes.

### Trial status

The trial is currently enrolling participants, starting from February 9, 2024, with recruitment expected to continue for about 2 years. We are following protocol version 3.1.0, dated October 13, 2023.

## Supplementary Information

Suplementary Material 1.

Suplementary Material 2.

Suplementary Material 3.

## Data Availability

The principal investigators, NA and YdK, will have access to the final dataset. Data will be available on reasonable request.

## Abbreviations

BBG: Blue light blocking glasses
BLT: Bright Light Therapy
BOLD: Blood-oxygenation level dependent
CEQ: Credibility/Expectancy Questionnaire
CSD: Consensus Sleep Diary
CSO: Current Sleep Onset
DLMO: Dim light melatonin onset
DTI: Diffusion Tensor Imaging
DWI: Diffusion Weighted Imaging
DMN: Default Mode Network
EMA: Ecological Momentary Assessment
FA: Fractional Anisotropy
FOV: Field of View
FPN: Fronto-Parietal Network
GM: Gray Matter
ISI: Insomnia Severity Index
LGI: Local Gyrification Index
MADRS: Montgomery Åsberg Depression Rating Scale
µMCTQ: Ultra short Munich ChronoType Questionnaire
MD: Mean diffusivity
MEQ: Morningness-Eveningness Questionnaire
MHQoL: Mental Health Quality of Life
M.I.N.I.: Mini Mental State Examination
MRI: Magnetic Resonance Imaging
PSQI: Pittsburg Sleep Quality Index
QIDS-SR: Quick Inventory of Depressive Symptomatology, Self-report
SA: Surface Area
SAD: Seasonal Affective Disorder
AE: Adverse Event
SN: Salience Network
SMN: Sensorimotor Network
TE: Echo time
THK: Thickness
TR: Repetition time
TSO: Target Sleep Onset
VOL: Volume
WM: White Matter
YMRS: Young Mania Rating Scale
