# Supplementary material for: BioClock—optimizing Bright Light Therapy for adults with depression: a study protocol for a multicenter randomized clinical trial on treatment strategies, response predictors, and chronobiological and neurobiological mechanisms
Source: Trials. 2025 Oct 14;26:411. doi: 10.1186/s13063-025-08984-7 (PMC12523188; doi:10.1186/s13063-025-08984-7)
Supplement: Supplementary file 1 — Suplementary Material 1. [file 13063_2025_8984_MOESM1_ESM.pdf]

CONTENT ECOLOGICAL MOMENTARY ASSESSMENT

**Variable**	**Questions**	**Answer options**
Positive Affect	I feel cheerful<br>I feel content<br>I generally feel well	Likert scale from 1 (*not at all*) to 7 (*very*)
Negative Affect	I feel insecure<br>I feel irritated<br>I feel lonely<br>I feel anxious<br>I feel down<br>I feel guilty	Likert scale from 1 (*not at all*) to 7 (*very*)
Vitality	I feel energetic<br>I feel lively<br>I feel active<br><br>Karolinska Sleepiness Scale:<br>How alert or sleepy do you feel right now?	Likert scale from 1 (not at all) to 7 (very/veel)<br><br>9 levels (1 = extremely alert, 2 = very alert, 3 = alert, 4 = rather alert, 5 = neither alert nor sleepy, 6 = some signs of sleepiness, 7 = sleepy, but no effort to keep awake, 8 = sleepy, some effort to keep awake, 9 = very sleepy, great effort keeping awake, fighting sleep). The scale includes labels on every second step (1, 3, 5, 7, and 9).
Context	What am I doing<br><br>Where are you right now?<br><br>With who are you right now?	Work, Study, Housekeeping, self-care, care for others, eating/drinking, traveling on the road, having a conversation, having online contact, sports, leisure, resting, other<br><br>Categories: “at home”, “at school”, “at work” “outside, in a public space”, “inside, in a public space” “at someone else’s home”, “other, being:”, “on the road”, “in nature”<br><br>Partner, Children, Pets, Housemates, Family, Friends, Colleages, Healthcare Workers, Acqaintances, Strangers or others, Nobody
**Variable**	**Questions**	Answer options

Sleep	What time did I go into bed?<br>What time did I try to go to sleep?<br>How long did it take me to fall asleep?<br>How many times did I wake up, not counting my final awakening?<br>In total, how long did these awakenings last?<br>What was the time of my final awakening?<br>What time did I get out of bed for the day?<br>How would I rate the quality of my sleep?<br>After my final awakening, how long did I spend in bed trying to sleep?<br>Did I wake up earlier than I planned?<br>How much earlier?<br>How rested or refreshed did I feel when I woke up?	Time<br>Time<br>Minutes<br>Number<br>Minutes<br>Time<br>Time<br>Scale from -3 (very bad) to +3 (very good)<br>Minutes<br>Yes/no Minutes<br>Likert scale from *not* to *very*<br>Scale from -3 (very bad) to +3 (very good)
**Variable**	**Questions**	Answer options
Sleep	How many times did I nap or doze?<br>In total, how long did I nap or doze?<br>How many drinks containing alcohol did I have<br>What time was the last alcoholic drink?<br>How many caffeinated drinks (coffee, tea, soda, energy drinks) did I have?<br>What time was my last caffeinated drink?<br>Did I take any over-the-counter or prescription medication(s) to help me sleep?	Number<br>Minutes<br>Number<br>Time<br>Number<br>Time<br>Yes/no
Treatment adherence	I received light therapy today<br>If no → Why did you not receive light therapy today?<br>If yes → how did you experience light therapy today?	Yes/No<br>"I forgot", "I had another appointments", It is uncomfortable", "It was not scheduled"<br>5 point likert scale from 0 (unpleasant) to 5 (very pleasant)
