# Supplementary material for: BioClock—optimizing Bright Light Therapy for adults with depression: a study protocol for a multicenter randomized clinical trial on treatment strategies, response predictors, and chronobiological and neurobiological mechanisms
Source: Trials. 2025 Oct 14;26:411. doi: 10.1186/s13063-025-08984-7 (PMC12523188; doi:10.1186/s13063-025-08984-7)

Monitoring in the BioClock study

Dear participant!

This is the user manual for the sensors and telephone monitoring in the BioClock study!

We are glad you are participating, and we will explain to you step by step how to wear and install the sensors correctly. It is essential to follow these steps carefully so that we can collect valuable data about your mood, biological clock, sleep-wake rhythm, light exposure, and energy levels throughout the day.

At the end of the measurement period, we will discuss all the results with you. We hope to tell you then how your lifestyle has changed during the therapy and how this has affected your mood. The better you wear the sensors and answer the questions in the app, the more valuable the participation in our study will be for you.

Please do not hesitate to contact our research team if you have any questions or encounter any difficulties during the process. We are here to help you! You can reach us at BioClock.Eindhoven@gmail.com

Thank you for your participation, and we hope you will have fun during this BioClock study!

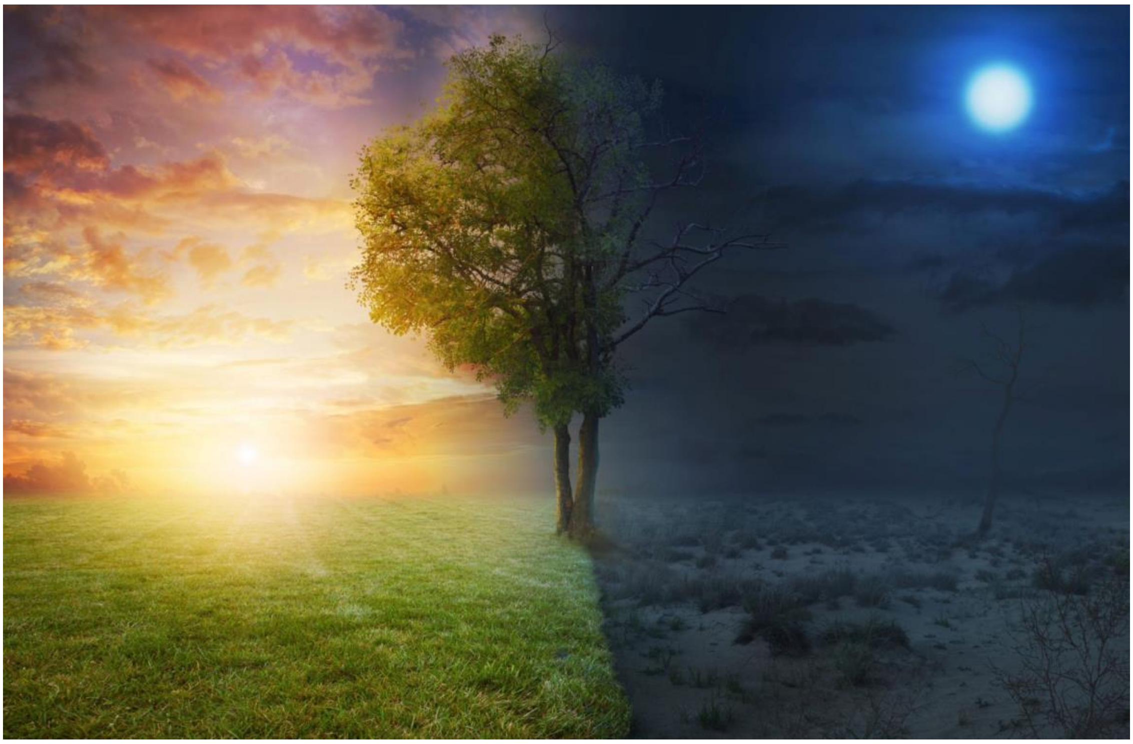

Sensor package contents

You will receive a box from the researchers that contains all the sensors. Check that the following items are present in the box:

- A black watch: This is the motion sensor. (See photo 1)
- A beige box with a clip attached: This is the light logger. (See photo 2)
- A piece of paper with a participant number.

Are you missing any of these parts? If so, please send an email to BioClock.Eindhoven@gmail.com

All the sensors in the box are programmed by the researcher to start measuring at the right time. So, you don't have to turn anything on before you can start wearing them.

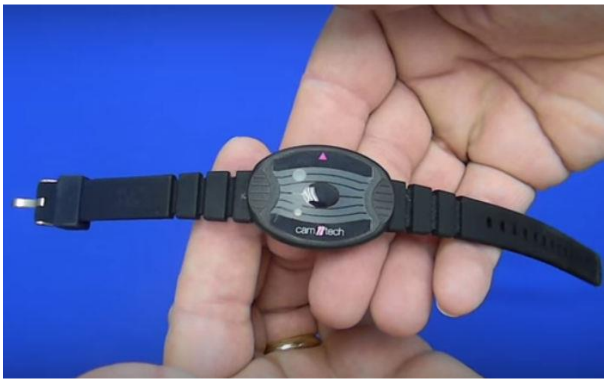

Photo 1: Motion sensor

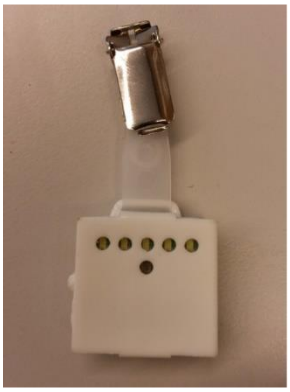

Photo 2: Light logger

Wearing the motion sensor

The motion sensor allows us to see how much you move. But that's not all! We can also watch when you go to sleep and wake up again because, during sleep, you move a little less. That way, we can find out if there are changes in your sleep and activity during therapy.

The watch is on 24 hours a day. Therefore, at the end of the measurement period, we can give you a good overview of your movement and sleep patterns. We can even give you tips to improve them and become healthier.

Here are a few important things to watch out for:

- Wear the watch on the other hand than the one you are writing with. So, if you are right-handed, wear the watch on your left wrist.
- The watch should be worn at all times, even while sleeping! This is because it also tracks your sleep activity.
- The watch is waterproof! You can shower with it and even swim with it.
- Sometimes, the strap may irritate a bit after long wear. If that happens, you can take the watch off for an hour. Please do let us know if you do.

Wearing the light logger

The light logger is a sensor that measures how much light is around you. The amount of light you receive during the day can affect your mood! We would like to find out if this is the case with you! To properly measure light, we would like to ask you to follow the rules below:

- Wear the light logger above your clothes at the level of your collarbone (in Photo 3, we have indicated where that is).
- Attach the light logger to your clothes with the metal clip. Important: do not use the magnetic clip! If you loosen it, the sensor may fall out of the box.
- The sensors that measure light are behind the six holes in the front of the light logger. Make sure these holes are not covered, for example, by your clothes or hair.
- Wear the sensor during the day. When you go to sleep, feel free to take the sensor off. Just put it upside down so no light is measured.
- The sensor is not waterproof. So, if it's raining outside, take the sensor off. Please do let us know when you have done so.

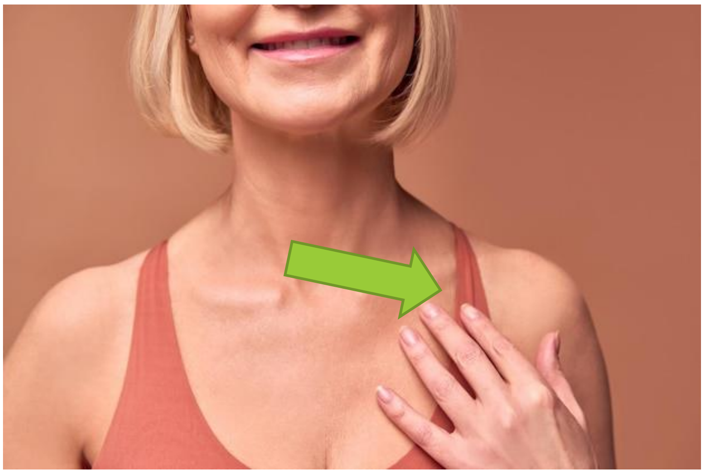

Photo 3: Try wearing the light sensor above your clothes at the level of the green arrow

Smartphone monitoring

We use telephone monitoring to track changes in your mood, sleep, and behavior throughout the day. We want to discover when you feel your best and what activities energize you. We will also compare your mood and behavior with the data we have collected with the motion sensor.

To participate in this monitoring, you need to install the M-Path app on your smartphone and add the BioClock study to your account. Below, we explain step by step how to do that.

Installing the app

To begin monitoring, you must first download and install the M-Path app on your smartphone. This app is available in the App Store for Apple users and on Google Play for Android users. Photo 4 shows an image of M-Path in the App Store and Google Play.

Don't have a smartphone? No worries! You can also use an iPad or another Android tablet.

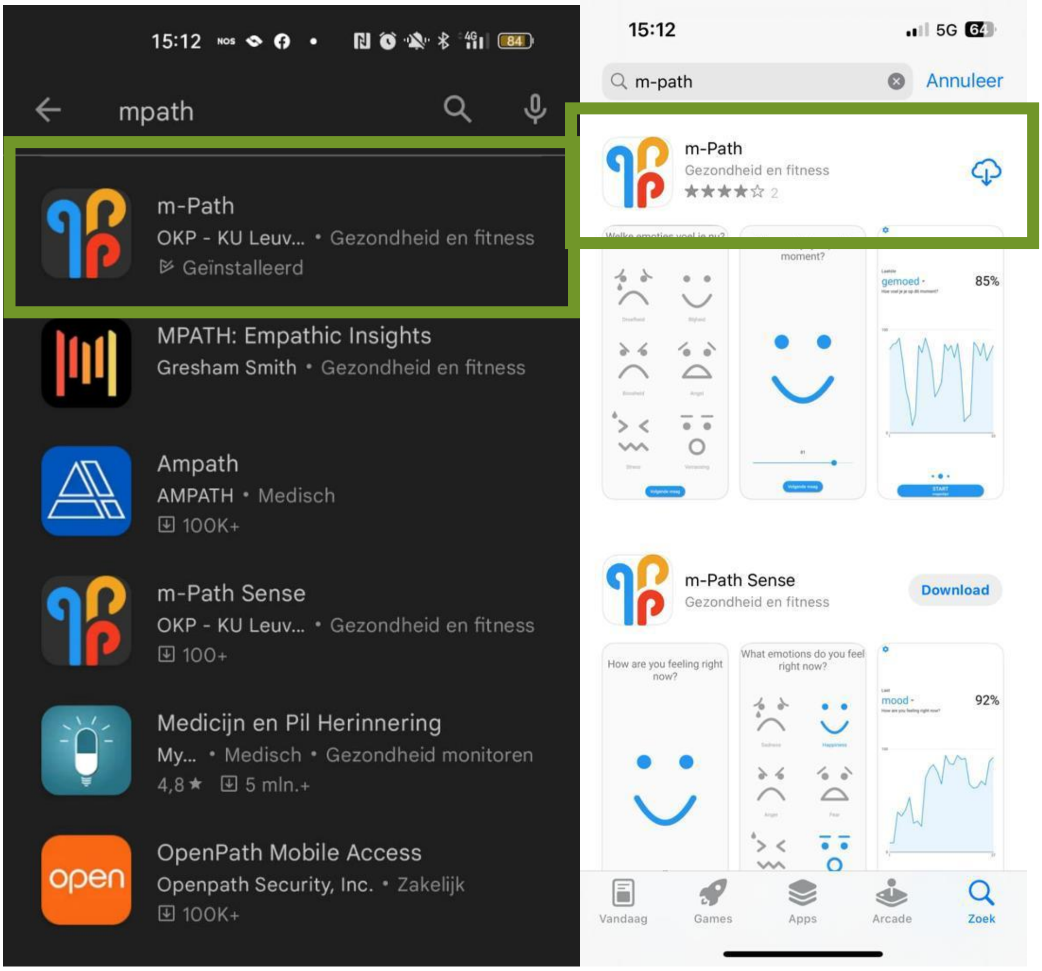

Photo 4: The app to download for telephone monitoring is indicated in green.

Create an account

Once you have downloaded M-Path, you can create an account. When you open the app, it asks you to enter an alias (see photo 5). To do this, use the **participant number** that was in the box you received from us. This is very important because we won't know who you are otherwise!

Once you have done this, you will come to a screen with the privacy terms (see photo 5). You must accept these to participate in the telephone monitoring. If there are things in them that you would rather not accept, send an e-mail to BioClock.Eindhoven@gmail.com

Your account has now been created! In order to restore your account on another phone or monitor it again next time, you need the recovery code. Write it down below so you don't lose it.

Recovery code: ....

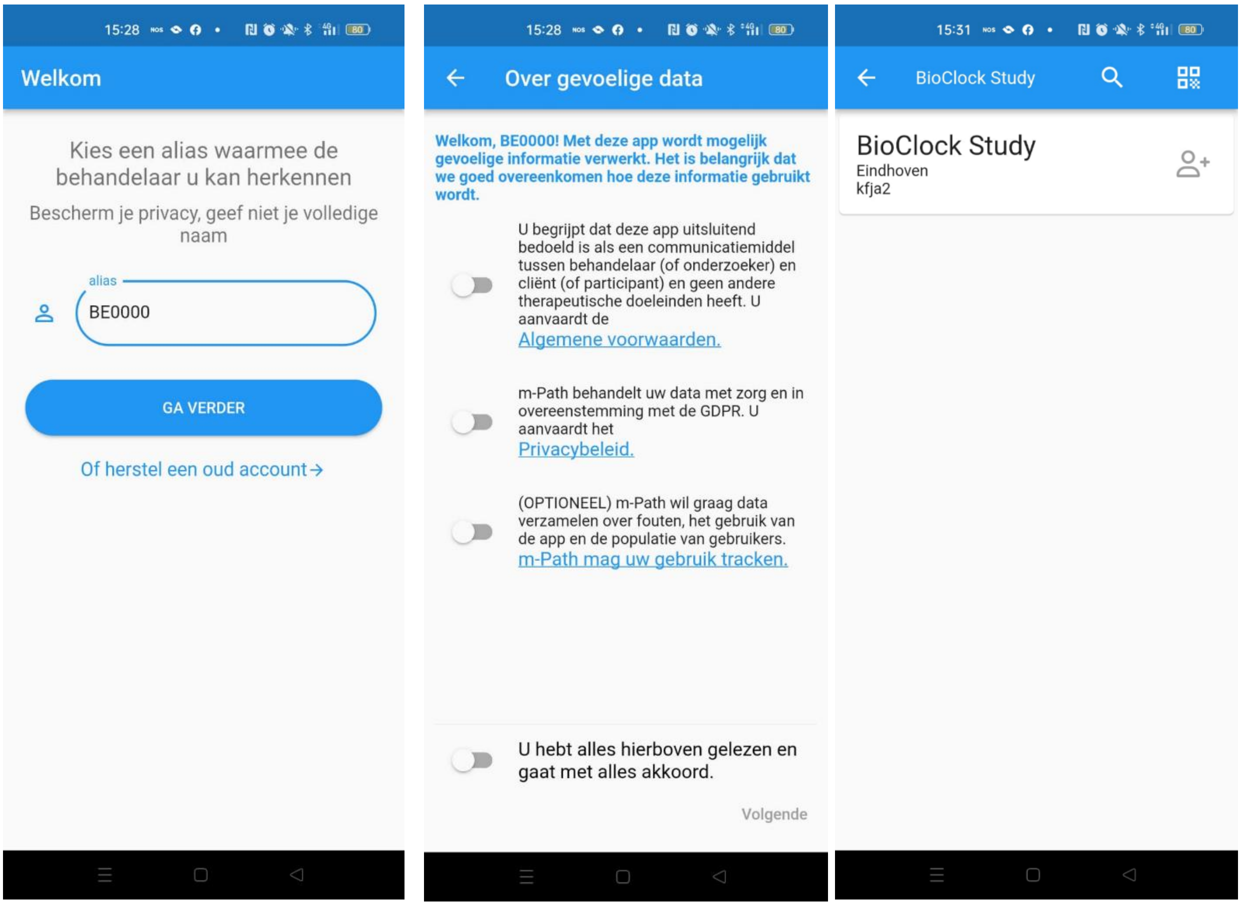

Photo 5: You will encounter these screens while creating an M-Path account and adding the BioClock study. On the left, you see how to enter an alias. In the middle are the privacy terms, on the right, how to add BioClock to your account.

Adding the BioClock monitoring

To add the BioClock study, simply search for "BioClock Study" in the search bar at the top of the screen. Once you find the right study, click on the icon with the plus sign next to it to add it.

From now on, you can start receiving notifications! The researcher should have told you when to expect them. So, keep a close eye on your phone!

How does monitoring work

The M-Path app will beep (and vibrate) 8 times at random during the day, between 8:00 am and 10:00 pm. At that time, you are supposed to fill out a questionnaire. Don't worry; filling it out only takes about 1 minute. In addition, in the morning and in the evening, you will receive a slightly longer questionnaire about your sleep and behaviors that may affect your sleep.

Try not to think too long about each question; just fill in what comes to mind. It is about what you thought, felt, and did, who you were with, and where you were just before the beep sounded. Take the time to fill in the questions calmly and accurately so that no mistakes are made. The better you do this, the more insight we will gain into your feelings and behavior, and the better we can help you improve your health.

The idea is not to stay awake or get up early to avoid missing the beeps. It is important just to keep your own rhythm. When you want to sleep, put your smartphone somewhere you can't hear it (for example, in the living room or kitchen) or just turn it off. That way, you can enjoy your sleep undisturbed.

Questions?

This is all the information you need about our monitoring measures within this study. If you still have questions, find something unclear, or have comments, please let us know at BioClock.Eindhoven@gmail.com

We hope that with this help, you can do the monitoring as well as you can. Tracking and linking your lifestyle changes to your mood can be important in your recovery! We wish you good luck and a positive experience with participating in this study!

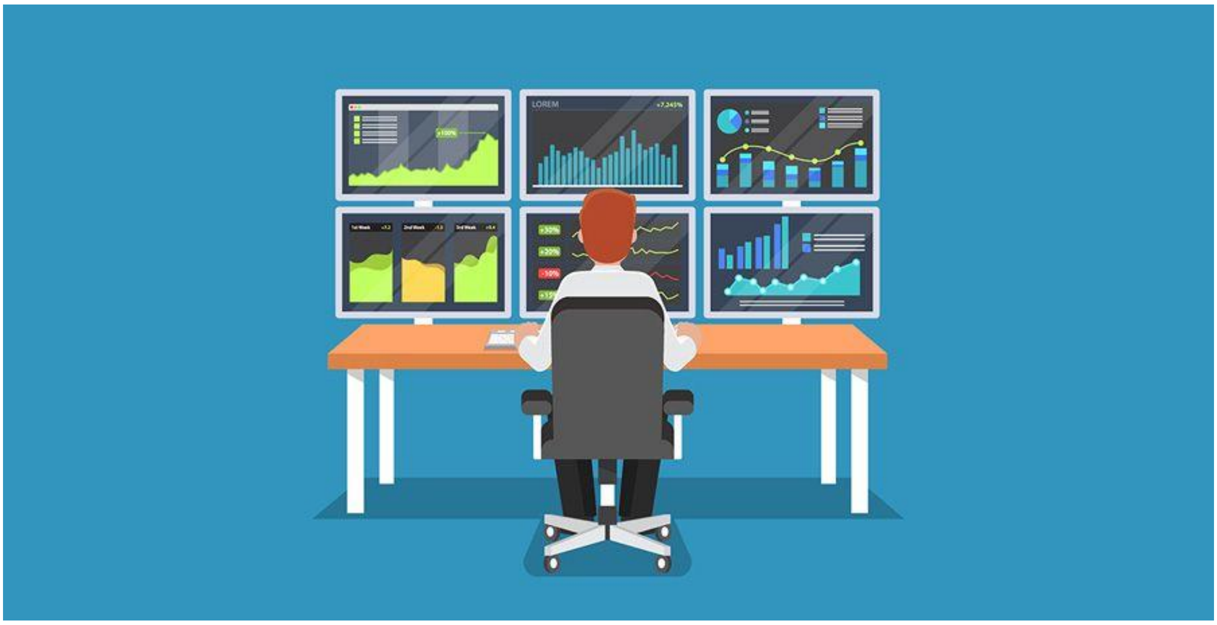

Supplement: Supplementary file 2 — Suplementary Material 2. [file 13063_2025_8984_MOESM2_ESM.pdf]
