# Supplementary material for: BioClock—optimizing Bright Light Therapy for adults with depression: a study protocol for a multicenter randomized clinical trial on treatment strategies, response predictors, and chronobiological and neurobiological mechanisms
Source: Trials. 2025 Oct 14;26:411. doi: 10.1186/s13063-025-08984-7 (PMC12523188; doi:10.1186/s13063-025-08984-7)
Supplement: Supplementary file 3 — Suplementary Material 3. [file 13063_2025_8984_MOESM3_ESM.pdf]

Chrono@Work

Research, Training & Consultancy

# INSTRUCTIONS FOR THE PARTICIPANT

## General instructions for the collection of saliva for melatonin analysis

MEDICATION

If you use medication regularly, please consult with the researcher- prior to this melatonin test - whether these medicines can be taken (normally) during the saliva collection. This is important because some medications can influence the melatonin test results.

LIGHT

From 1 hour before you take the first sample up to and including the last sample you must be in dimmed light (very little light). Close the curtains. If any light comes in you must wear sunglasses. This also applies if you leave that room, for example for a toilet or toilet bathroom visit. Only illuminate the room with a weak table lamp at a great distance, it should be just enough to be able to read. You can watch TV at a distance of at least 3 meters. Do not sit closer to the TV or look into the lamp. The light intensities of all screens that you use, e.g. smartphone, tablet and PC must be on the lowest light intensity.

BODY POSITION

You may take the samples while sitting or lying down, but you must not change your posture within 5 minutes prior to taking the saliva sample. So, make sure you have the tube lie next to you.

EATING & DRINKING

Drinking water or fruit juices or eating is allowed after taking a saliva sample, but not in the half hour prior to collecting the next saliva sample. Coffee, tea, chocolate, bananas and foods with coloring agents (e.g. candy) are not allowed. Smoking may be allowed during this time, but please try to smoke as little as possible. You may brush your teeth with water but not with toothpaste. Only after taking the last sample you can brush with toothpaste.

MOUTH RINSING

After eating or drinking and in any case at least 10 minutes before the next saliva sample always rinse your mouth with water (this also applies prior to the first sample in the evening).

INSTRUCTIONS SALIVA COLLECTION

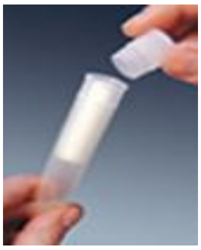

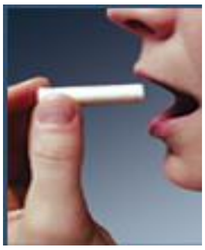

Click the cap off the tube and put the cotton swab in your mouth. Hold the swab there for a few minutes until it gets quite wet and then put it back in the tube (upper part). Press the cap back on the tube firmly. Write the time on the supplied code form. You can now eat / drink something (see above) and then rinse your mouth with water. Make sure the cotton swab is in the upper part of the tube.

SALIVA COLLECTION TIME POINTS

Pay close attention to when you have to start collecting saliva! This is 4 hours before you generally go to bed. This also means that that evening, from 5 hours before you go to bed, you have to spend in very little light. So plan that day well in advance to make sure that you follow all the instructions!

ATTENTION, keep the tubes in the refrigerator until you send them back. At night you do not have to take every tube to the fridge but you can do this in the morning after waking up. Please sen~~t~~d the tubes to u~~a~~s as soon as possible. More information about how to do this can be found in the letter. The envelope can simply be placed in the orange PostNL mailbox.

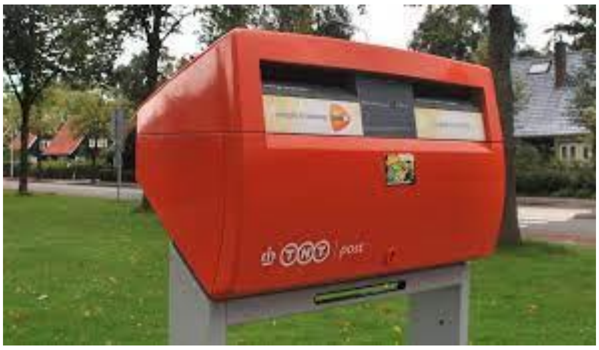

Our privacy statement applies to your data. You can find this statement on our website www.chronoatwork.com.
